# A Simple and Efficient Synthesis of Highly Substituted Indeno[1,2-*b*]pyrrole and Acenaphtho[1,2-*b*]pyrrole Derivatives by Tandem Three-Component Reactions

**DOI:** 10.3390/molecules23113031

**Published:** 2018-11-20

**Authors:** Xiaodong Tang, Songlei Zhu, Ying Ma, Ren Wen, Lanqi Cen, Panwei Gong, Jing Wang

**Affiliations:** 1 Department of Chemistry, School of Pharmacy, Xuzhou Medical University, Xuzhou 221004, Jiangsu, China; windmillons@126.com (X.T.); yingma123@126.com (Y.M.); lanqi.cen@siat.ac.cn (L.C.); g1578989778@163.com (P.G.); 2 Nanjing Hicin Pharmaceutical Co., Ltd., Nanjing 210009, Jiangsu, China; 15950589107@163.com

**Keywords:** multi-component reactions, indeno[1,2-*b*]pyrroles, acenaphtho[1,2-*b*]pyrroles

## Abstract

A green, convenient and tandem procedure for the efficient synthesis of highly substituted indeno[1,2-*b*]pyrrole and acenaphtho[1,2-*b*]pyrrole derivatives by domino three-component reaction of tryptamine/benzylamine, 1,3-dicarbonyl compounds and ninhydrin/ acenaphthenequinone is described. The significant features of this procedure were characterized by mild reaction conditions, high yields, operational simplicity and it being environmentally benign.

## 1. Introduction

**Figure 1:**
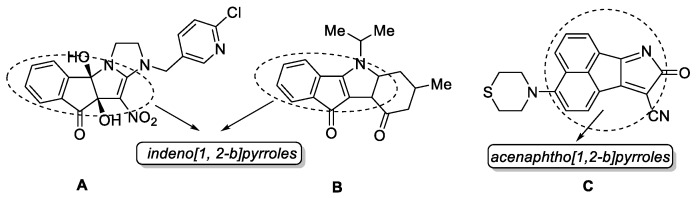
Biologically important indenopyrroles and acenaphthopyrroles.

Polysubstituted pyrroles are widely employed as versatile building blocks in synthetic organic chemistry [1,2,3,4] because of their presence in numerous natural products and drug molecules, and because they exhibit different pharmacological applications including anti-tuberculosis, anti-oxidation, antibacterial, anti-inflammatory, and antitumor properties, among others [5,6,7]. Among them, indeno[1,2-*b*]pyrroles (Figure 1A,B) are heterocycles of great importance because they can be used as antiviral agents, insecticides, herbicides and human protein kinase CK2 inhibitors [8,9,10]. Moreover, acenaphtho[1,2-*b*]pyrroles (Figure 1C), as important polycyclic fused compounds, can also be employed as potent and selective inhibitors of fibroblast growth factor receptor 1 (FGFR-1), novel Bcl-2 inhibitors, and as a valuable Mcl-1 inhibitor [11,12,13,14].

The development of the design and synthesis of diverse heterocyclic compounds with valuable medicinal and biological applications is highly desirable in current organic and medicinal chemistry research [15,16,17,18]. Multi-component reactions (MCRs), which involve the rapid combination of three or more simple reactants in a one-pot sequential process and produce the final product containing a substructure of all starting materials, play an important role in the synthesis of complex and diverse molecules [19,20,21]. MCRs have attracted much attention for the construction of bioactive heterocyclic compounds, due to their high productivity, facile execution, convergence, low costs, minimal waste production and structural diversity [22,23,24,25,26,27,28].

Considering the importance of indeno[1,2-*b*]pyrrole and acenaphtho[1,2-*b*]pyrrole derivatives and in continuation of our research on multi-component reactions [29,30,31,32], herein we report a three-component reaction of ninhydrin/acenaphthenequinone, 1,3-dicarbonyl compounds and tryptamine/benzylamine, which is an efficient and straightforward protocol for the synthesis of a serial of highly substituted indeno[1,2-*b*]pyrrole and acenaphtho[1,2-*b*]pyrrole derivatives.

## 2. Results and Discussion

**Scheme 1:**
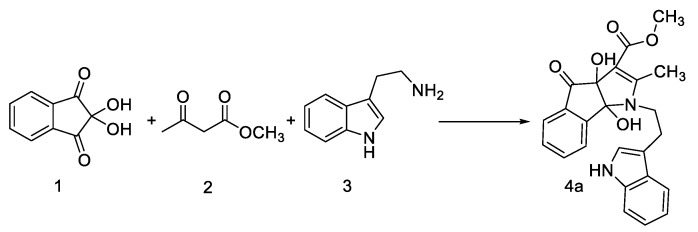
Model reaction.

Initially, we carried out the one-pot, three-component reaction of ninhydrin **1**, methyl acetoacetate **2**, and tryptamine **3** as a model reaction to establish the feasibility of the strategy and optimize reaction conditions (Scheme 1). The reaction was examined in different solvents including methanol, ethanol, chloroform, acetonitrile, toluene and water. As shown in Table 1, using ethanol as the solvent provided the highest yield (Table 1, Entry 7). Furthermore, the reaction was carried out at different temperatures, ranging from room temperature to refluxing. It can be seen from Table 1 that temperature had no remarkable effect on this reaction. Therefore, using ethanol as the solvent, and carrying out the reaction at room temperature, were chosen as optimal conditions for all further reactions.

**Scheme 2:**
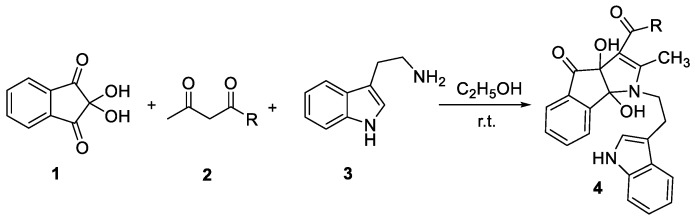
The synthesis of compounds **4**.

**Scheme 3:**
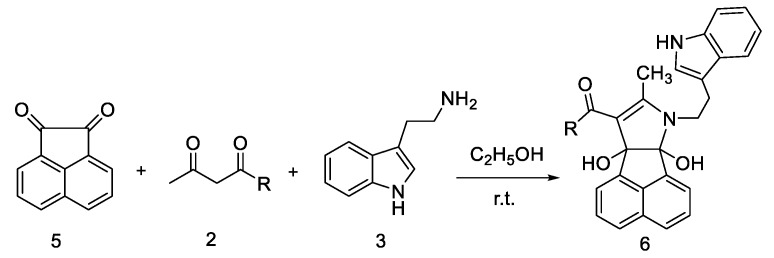
The synthesis of compound **6**.

With optimum conditions determined, we explored the model reaction using different 1,3-dicarbonyl compounds with ninhydrin and tryptamine (Scheme 2). As shown in Table 2, the reaction performed smoothly in 2.5–3.5 h, with excellent yields of 84–94%. In order to expand the scope of this protocol, ninhydrin was replaced by acenaphthenequinone to react with different 1,3-dicarbonyl compounds and tryptamine (Scheme 3). To our delight, a new series of acenaphtho[1,2-*b*]pyrrole derivatives were obtained easily with satisfactory yields (Table 3).

**Scheme 4:**
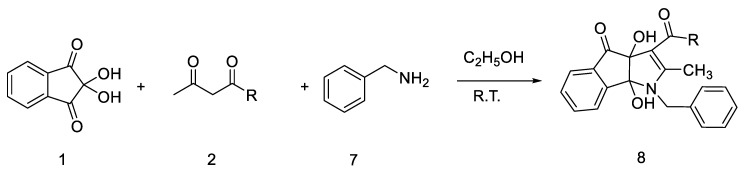
The synthesis of compounds **8**.

To further extend the usefulness of this methodology, the one-pot reactions of ninhydrin and 1,3-dicarbonyl compounds with benzylamine **7** were also investigated (Scheme 4), and the results are presented in Table 4.

**Scheme 5:**
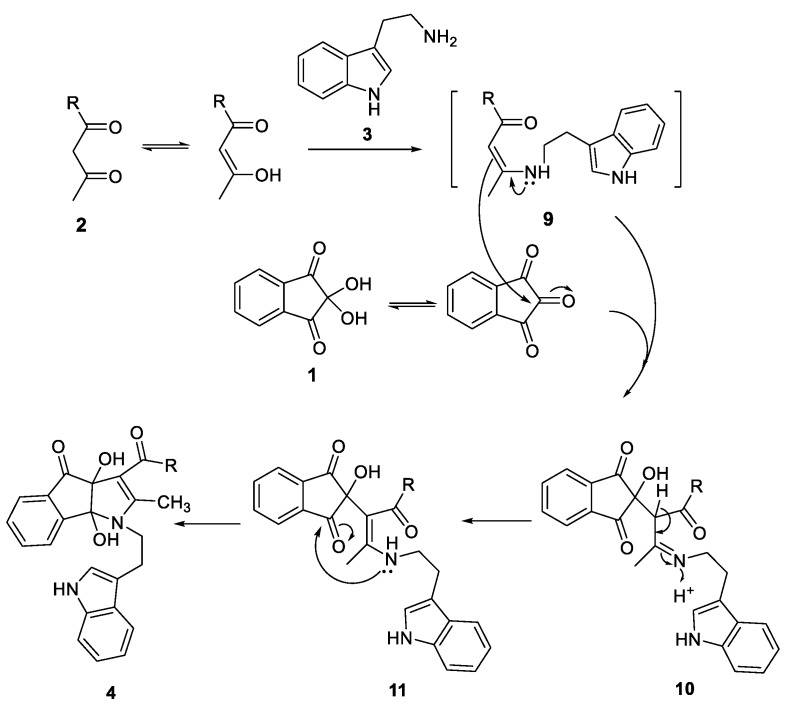
Proposed mechanism for the formation of **4**.

A reasonable mechanism of the reaction is given in Scheme 5. Initially, the reaction between acetoacetate **2** and tryptamine **3** is to give intermediate enamine **9**, which further undergoes the nucleophilic addition with the carbonyl in ninhydrin **1** to afford the intermediate **10**. After isomerization of **10** to the aminol intermediate **11**, the subsequent intermolecular *N*-cyclization afforded the target product **4**.

To verify this conversion, we carried out the model reaction with three reactants mixed simultaneously in ethanol solvent at room temperature. The target product was also formed, although only a 53% yield of **4a** was obtained. When acetoacetate **2** and tryptamine **3** were pre-stirred and ninhydrin **1** was added subsequently under one-pot reaction conditions without any separation, the purpose product was obtained with a satisfactory yield of 85%. Therefore, this three-component reaction might be accomplished by a tandem sequential procedure, which the first condensation of 1,3-dicarbonyl compound and amine to afford intermediate enamine may facilitate to achieve better results.

**Figure 2:**
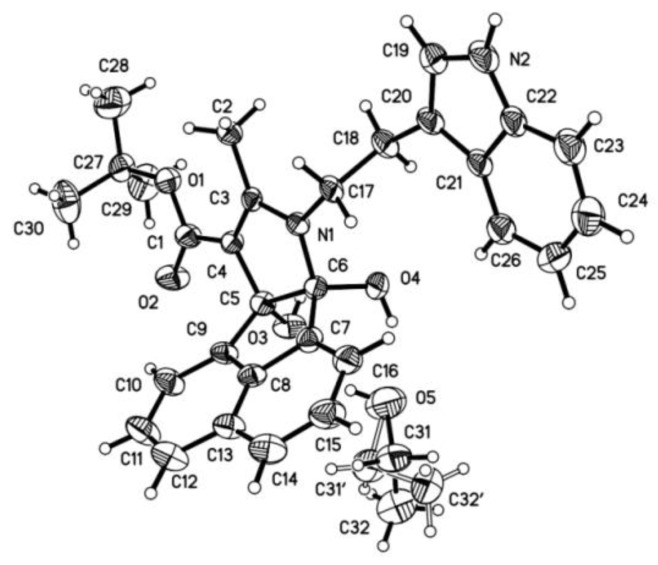
The crystal structure of **6d** with ethanol solvent.

**Figure 3:**
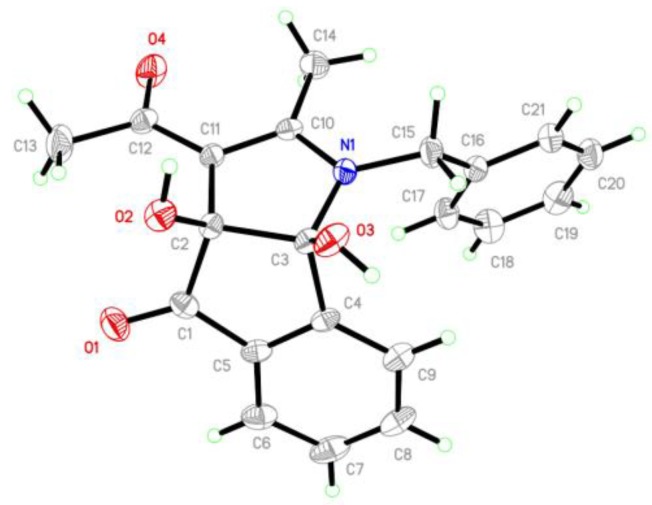
The crystal structure of **8f**.

In this study, all products were characterized by melting point, IR, NMR, and HRMS spectral data (All the data were in the supplementary). The ^1^H-NMR spectrum of compound **4a** showed two singlets at 2.22 and 3.55 ppm, which are distributed to one methyl group and one methoxyl group, respectively. Four multiplets appeared at the 2.92 to 4.06 ppm area and are related to the four protons of two linked methylene moieties. The sharp single signals at 5.67 and 6.77 were assigned to two hydroxyl groups. The aromatic protons resonated between 7.02 and 7.89, and the NH proton of indole was observed at 10.92 ppm. Furthermore, the structures of compounds **6d** (Figure 2) and **8f** (Figure 3) were also confirmed by X-ray crystallographic analysis. In the crystal structure of **6d**, the dihedral angle was formed between the acenaphthequinone plane and pyrrole plane, while in the structure of **8f** this was between the indeno plane and pyrrole plane. Therefore, the two adjacent OH groups which attached to the fused side of two planes might adopt cis configuration. Furthermore, a solvent molecule of ethanol was involved in the single crystal unit of **6d** to form co-crystal. 

## 3. Materials and Methods

### 3.1. General Information

Melting points were recorded on an Electrothermal digital melting point apparatus (Donghua, Shanghai, China) and were uncorrected. IR Spectra were recorded on a Nicolet FT-IR500 spectrophotometer (Madison, WI, USA) using KBr optics. ^1^H-NMR and ^13^C-NMR spectra were recorded on a JMTC-400/54/SS spectrometer (JEOL Ltd., Tokyo, Japan) using DMSO-*d*_6_ as solvent and TMS as internal standard. HRMS analyses were conducted on a Bruker micro-TOF-Q-MS analyzer (Bruker Daltonics, Bremen, Germany). X-Ray diffraction data were made on a Rigaku Mercury CCD area detector with graphite monochromated Mo-Ka radiation (Rigaku, Tokyo, Japan).

### 3.2. General Procedure for the Synthesis of Compounds ***4***, ***6*** and ***8***

A mixture of an equimolar amount of 1,3-dicarbonyl compound **2** (0.5 mmol) and tryptamine **3** (0.5 mmol) or benzylamine **7** (0.5 mmol) were stirred in ethanol at room temperature for 1 h. Ninhydrin **1** (0.5 mmol) or Acenaphthenequinone **5** (0.5 mmol) was then added to the solution and stirred at room temperature for 1.5–2 h. Completion of the reaction was monitored with TLC. The mixture was poured into cold water. The precipitate was filtered and washed with EtOH (95%). The precipitate was purified by recrystallization from EtOH to give the products **4**, **6** and **8**, respectively.

*Methyl 1-(2-(1H-indol-3-yl)ethyl)-3a,8b-dihydroxy-2-methyl-4-oxo-1,3a,4,8b-tetrahydroindeno[1,2-b]pyrrole-3-carboxylate* (**4a**), yellow soild; m.p. 149–151 °C; IR (cm^−1^): 744, 1194, 1356, 1437, 1551, 1655, 1717, 3410; ^1^H-NMR (400 MHz, DMSO-*d*_6_; δ, ppm): 2.22 (s, 3H, CH_3_), 2.92–3.00 (m, 1H, CH), 3.14–3.21 (m, 1H, CH), 3.55 (s, 3H, OCH_3_), 3.72–3.80 (m, 1H, CH), 3.98–4.06 (m, 1H, CH), 5.67 (s, 1H, OH), 6.77 (s, 1H, OH), 7.02 (t, *J* = 8.0 Hz, 1H, ArH), 7.10 (t, *J* = 8.0 Hz, 1H, ArH), 7.29 (s, 1H, ArH), 7.37 (d, *J* = 8.0 Hz, 1H, ArH), 7.56 (t, *J* = 8.0 Hz, 1H, ArH), 7.63 (d, *J* = 8.0 Hz, 1H, ArH), 7.70–7.77 (m, 2H, ArH), 7.89 (d, *J* = 8.0 Hz, 1H, ArH), 10.92 (s, 1H, NH); ^13^C-NMR (100 MHz, DMSO-*d*_6_; δ, ppm): 13.16, 27.45, 43.10, 50.15, 84.97, 94.74, 95.15, 111.74, 111.99, 118.86, 118.97, 121.56, 123.50, 123.72, 124.96, 127.62, 130.64, 135.51, 136.01, 136.73, 148.68, 160.53, 166.27, 198.83. HRMS calcd. for C_24_H_22_N_2_O_5_ [M + H]^+^: 418.1529, found: 418.1545.

*Methyl 7-(2-(1H-indol-3-yl)ethyl)-6b,9a-dihydroxy-8-methyl-6b,9a-dihydro-7H-acenaphtho-[1,2-b]pyrrole-9-carboxylate* (**6a**), white soild; m.p. 161–163 °C; IR (cm^−1^): 746, 787, 833, 1003, 1080, 1198, 1383, 1439, 1560, 1637, 3421; ^1^H-NMR (400 MHz, DMSO-*d*_6_; δ, ppm): 2.19 (s, 3H, CH_3_), 2.84–2.91 (m, 1H, CH), 3.06–3.13 (m, 1H, CH), 3.66 (s, 3H, CH_3_), 3.70–3.76 (m, 2H, CH_2_), 5.51 (s, 1H, OH), 6.49 (s, 1H, OH), 6.99 (t, *J* = 8.0 Hz, 1H, ArH), 7.07 (t, *J* = 8.0 Hz, 1H, ArH), 7.25 (s, 1H, ArH), 7.34 (d, *J* = 8.0 Hz, 1H, ArH), 7.52–7.62 (m, 4H, ArH), 7.70 (d, *J* = 8.0 Hz, 1H, ArH), 7.76–7.82 (m, 2H, ArH), 10.90 (s, 1H, NH); ^13^C-NMR (100 MHz, DMSO-*d*_6_; δ, ppm): 13.05, 19.11, 27.00, 43.19, 50.08, 56.58, 87.65, 99.60, 100.90, 111.96, 112.03, 118.75, 118.86, 119.60, 121.57, 123.58, 123.85, 125.27, 127.62, 128.27, 129.10, 131.30, 136.45, 136.75, 142.28, 145.98, 160.48, 166.46. HRMS calcd. for C_27_H_24_N_2_O_4_ [M + H]^+^: 440.1736, found: 440.1745.

*Methyl 1-benzyl-3a,8b-dihydroxy-2-methyl-4-oxo-1,3a,4,8b-tetrahydroindeno[1,2-b]pyrrole-3-carboxylate* (**8a**), white soild; m.p. 154–156 °C; IR (cm^−1^): 784, 823, 1101, 1296, 1427, 1562, 1635, 1721, 3398; ^1^H-NMR (400 MHz, DMSO-*d*_6_; δ, ppm): 1.93 (s, 3H, CH_3_), 3.50 (s, 3H, CH_3_), 4.75 (d, *J* = 16.0 Hz, 1H, CH), 5.12 (d, *J* = 16.0 Hz, 1H, CH), 5.70 (s, 1H, OH), 6.79 (s, 1H, OH), 7.19–7.29 (m, 5H, ArH), 7.53 (t, *J* = 8.0 Hz, 1H, ArH), 7.65–7.70(m, 2H, ArH), 7.77 (d, *J* = 8.0 Hz, 1H, ArH); ^13^C-NMR (100 MHz, DMSO-*d*_6_; δ, ppm): 13.65, 45.38, 50.20, 85.04, 94.63, 95.59, 123.46, 125.27, 127.23, 127.37, 128.84, 130.65, 135.49, 135.86, 139.40, 148.54, 160.86, 166.19, 198.81. HRMS calcd. for C_21_H_19_NO_5_ [M + H]^+^: 365.1263, found: 365.1279.

## 4. Conclusions

In summary, we have developed a convenient three-component reaction for the preparation of some highly substituted indeno[1,2-*b*]pyrrole and acenaphtho[1,2-*b*]pyrrole derivatives in high yields. This protocol offers several advantages such as easy work-up, mild reaction times, as well as readily available starting materials, which makes it a useful and attractive process for the synthesis of the biologically important polysubstituted pyrroles.

## Figures and Tables

**Table 1:** Optimizing the reaction solvent for the synthesis of **4a**.

Entry	Solvent	Temp (°C)	Time (h)	Yield (%) ^a^
1	Methanol	r.t.	3	57
2	Chloroform	r.t.	3	40
3	Acetonitrile	r.t.	3	45
4	Toluene	r.t.	3	35
5	Water	r.t.	3	31
7	Ethanol	r.t.	3	85
8	Ethanol	40	3	83
9	Ethanol	reflux	3	84

^a^ Isolated yield.

**Table 2:** The synthesis of compounds **4**.

Entry	Product	Structure	Time (h)	Yield (%) ^a^	m.p./(°C)
1	**4a**	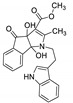	3	85	149–151
2	**4b**	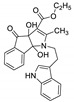	2.5	93	121–123
3	**4c**	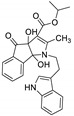	2.5	92	101–103
4	**4d**	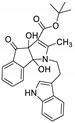	3	91	110–112
5	**4e**	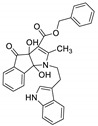	3.5	94	100–102
6	**4f**	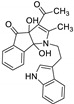	2.5	75	164–166

^a^ Isolated yield.

**Table 3:** The synthesis of compound **6**.

Entry	Product	Structure	Time (h)	Yield (%) ^a^	m.p./(°C)
1	**6a**	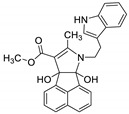	3	72	161–163
2	**6b**	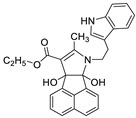	3	83	145–147
3	**6c**	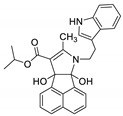	2.5	78	161–163
4	**6d**	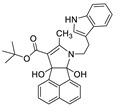	2.5	70	112–114
5	**6e**	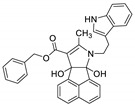	3.5	85	95–97

^a^ Isolated yield.

**Table 4:** The synthesis of compounds **8**.

Entry	Product	Structure	Time (h)	Yield (%) ^a^	m.p./(°C)
1	**8a**	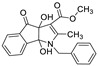	2.5	75	154–156
2	**8b**	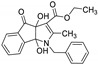	3	71	72–73
3	**8c**	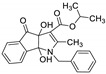	2.5	83	71–73
4	**8d**	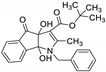	3.5	88	83–85
5	**8e**	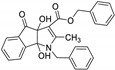	4	88	70–71
6	**8f**	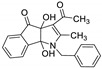	2.5	81	175–177

^a^ Isolated yield.

## Supplementary Materials

Supplementary data associated with this article are available online.
