# Supplementary material for: A Simple and Efficient Synthesis of Highly Substituted Indeno[1,2-b]pyrrole and Acenaphtho[1,2-b]pyrrole Derivatives by Tandem Three-Component Reactions
Source: Molecules. 2018 Nov 20;23(11):3031. doi: 10.3390/molecules23113031 (PMC6278260; doi:10.3390/molecules23113031)

SUPPORTING INFORMATION

A simple and efficient synthesis of highly substituted indeno[1,2-*b*]pyrrole and acenaphtho[1,2-*b*]pyrrole derivatives by tandem three-component reactions

Songlei Zhu [1,†], Xiaodong Tang [1,†], Ying Ma [1], Ren Wen [2], Lanqi Cen [1], Panwei Gong [1], and Jing Wang [1,*]

[1] Department of Chemistry, School of Pharmacy, Xuzhou Medical University, Xuzhou Jiangsu 221004, China;
[2] Nanjing Hicin Pharmaceutical Co.,Lt, Nanjing, Jiangsu 210009, China

*To whom correspondence should be addressed.
E-mail: songleizhu@xzmc.edu.cn
Phone: 86-516-83262098

Table of Contents

1. Experimental......1
    1.1. General......2
    1.2. Typical experimental procedure......2
2. X-Ray crystallographic data......3-4
3. Characterization data of compounds......5-11
4. Original ^{1}HNMR and ^{13}CNMR spectra for all compounds......12-28

1. Experimental

1.1. General

Melting points were recorded on an Electrothermal digital melting point apparatus and uncorrected. IR Spectra were recorded on a Nicolet FT-IR500 spectrophotometer using KBr optics. ^{1}H NMR and ^{13}C MNR spectra were recorded on a JMTC-400/54/SS spectrometer using DMSO-d_6 as solvent and TMS as internal standard. HRMS analyses were conducted on a Bruker micro-TOF-Q-MS analyzer. X-Ray diffraction data were made on a Rigaku Mercury CCD area detector with graphite monochromated Mo-Ka radiation.

1.2. Typical experimental procedure

1.2.1 Typical experimental procedure for **4**

A mixture of an equimolar amount of 1,3-dicarbonyl compound **2** (0.5 mmol) and tryptamine **3** (0.5 mmol) was stirred in ethanol at room temperature for 1h. Ninhydrin **1** (0.5 mmol) was then added to this solution and stirred at room temperature for 0.5-1h. Completion of the reaction was monitored with TLC, the mixture was poured into cold water, then filtered and washed with EtOH (95%). The precipitate was purified by recrystallization from EtOH to give the product **4**.

1.2.2 Typical experimental procedure for **6**

A mixture of an equimolar amount of 1,3-dicarbonyl compound **2** (0.5 mmol) and tryptamine **3** (0.5 mmol) was stirred in ethanol at room temperature for 1h. Acenaphthenequinone **5** (0.5 mmol) was then added to this solution and stirred at room temperature for 0.5-1h. Completion of the reaction was monitored with TLC, the mixture was poured into cold water, then filtered and washed with EtOH (95%). The precipitate was purified by recrystallization from EtOH to give the product **6**.

1.2.3 Typical experimental procedure for **8**

A mixture of an equimolar amount of 1,3-dicarbonyl compound **2** (0.5 mmol) and benzylamine **7** (0.5 mmol) were stirred in ethanol at room temperature for 2h. Ninhydrin **1** (0.5 mmol) was then added to this solution and stirred at room temperature for 0.5-1h. Completion of the reaction was monitored with TLC, the reaction mixture was poured into ice cold water. Then, the addition of brine solution

to the reaction mixture brought the precipitate. The precipitate was filtered, washed with water to give the product **8**. The crystal for X-ray diffraction was developed by recrystallization from EtOH.

2. X-Ray crystallographic data

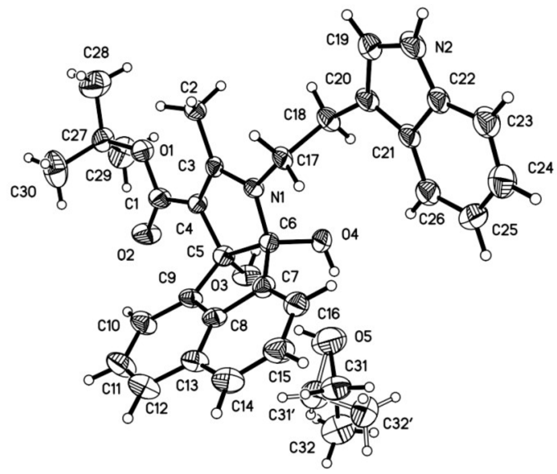

Figure 1.The crystal structure of **6d** with ethanol solvent

Table 1. Crystallographic data of compound **6d** (CCDC**:** 1866992)

Empirical formula	$C_{32}H_{36}N_2O_5$
Formula weight	528.63
Temperature	298(2) K
Wavelength	0.71073 A
Crystal system, space group	Triclinic, P-1
Unit cell dimensions	a = 9.9141(9) A alpha = 117.069(3) deg.<br>b = 12.5687(11) A beta = 93.6000(10) deg.<br>c = 13.6349(12) A gamma = 107.657(2) deg.
Volume	1400.7(2) A^3
Z, Calculated density	2, 1.253 Mg/m^3
Absorption coefficient	0.085 mm^-1
F(000)	564
Crystal size	0.30 x 0.18 x 0.14 mm
Theta range for data collection	2.35 to 25.02 deg.
Limiting indices	-11<=h<=11, -11<=k<=14, -16<=l<=15
Reflections collected / unique	6978 / 4822 [R(int) = 0.0878]
Data / restraints / parameters	4822 / 0 / 378
Goodness-of-fit on F^2	0.87
Final R indices [I>2sigma(I)]	R1 = 0.0941, wR2 = 0.2203
R indices (all data)	R1 = 0.2250, wR2 = 0.2842

Largest diff. peak and hole	0.483 and -0.279 e.A^-3

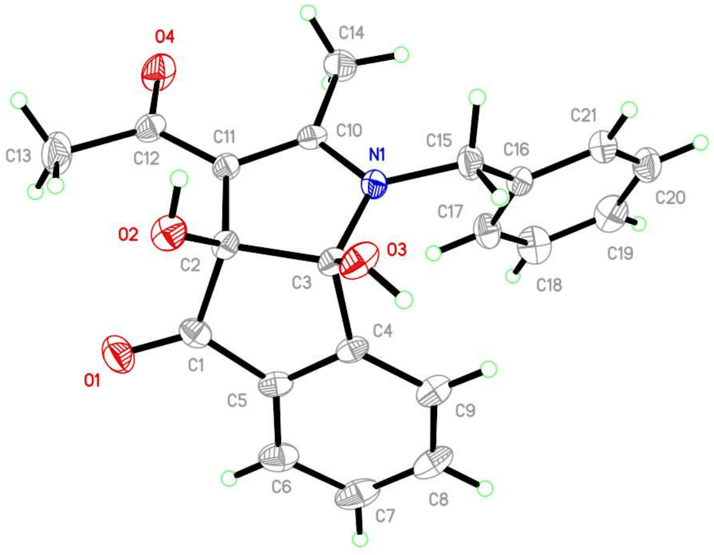

Figure 2.The crystal structure of **8f**

Table 2. Crystallographic data of compound **8f** (CCDC: 1866994)

Empirical formula	$C_{21}H_{19}NO_4$
Formula weight	349.37
Temperature	298(2) K
Wavelength	0.71073 A
Crystal system, space group	Monoclinic, P2(1)/n
	a = 8.1950(9) A alpha = 90 deg.
Unit cell dimensions	b = 16.7069(15) A beta = 102.369(2) deg.
	c = 12.8179(12) A gamma = 90 deg.
Volume	1714.2(3) A^3
Z, Calculated density	4, 1.354 Mg/m^3
Absorption coefficient	0.094 mm^-1
F(000)	736
Crystal size	0.45 x 0.21 x 0.18 mm
Theta range for data collection	2.44 to 25.02 deg.
Limiting indices	-9<=h<=8, -19<=k<=18, -15<=l<=15
Reflections collected / unique	8456 / 3003 [R(int) = 0.0420]
Data / restraints / parameters	3003 / 0 / 235
Goodness-of-fit on F^2	1.017
Final R indices [I>2sigma(I)]	R1 = 0.0465, wR2 = 0.1078
R indices (all data)	R1 = 0.0854, wR2 = 0.1269
Largest diff. peak and hole	0.246 and -0.241 e.A^-3

3. Characterization data of compounds

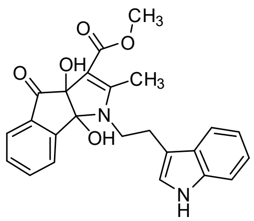

4a) ***methyl 1-(2-(1H-indol-3-yl)ethyl)-3a,8b-dihydroxy-2-methyl-4-oxo-1,3a,4,8b-tetrahydroindeno[1,2-b]pyrrole-3-carboxylate***

Yellow soild; m.p.: 149-151°C; IR(cm^{-1}): 744, 1194, 1356, 1437, 1551, 1655, 1717, 3410; ^1H NMR (400 MHz, DMSO-d_6; δ, ppm): 2.22 (s, 3H, CH_3), 2.92-3.00 (m, 1H, CH), 3.14-3.21 (m, 1H, CH), 3.55 (s, 3H, OCH_3), 3.72-3.80 (m, 1H, CH), 3.98-4.06 (m, 1H, CH), 5.67 (s, 1H, OH), 6.77 (s, 1H, OH), 7.02 (t, *J*=8.0 Hz, 1H, ArH), 7.10 (t, *J*=8.0 Hz, 1H, ArH), 7.29 (s, 1H, ArH), 7.37 (d, *J*=8.0 Hz, 1H, ArH), 7.56 (t, *J*=8.0 Hz, 1H, ArH), 7.63 (d, *J*=8.0 Hz, 1H, ArH), 7.70-7.77 (m, 2H, ArH), 7.89 (d, *J*=8.0 Hz, 1H, ArH), 10.92 (s, 1H, NH); ^{13}C NMR (100 MHz, DMSO-d_6; δ, ppm): 13.16, 27.45, 43.10, 50.15, 84.97, 94.74, 95.15, 111.74, 111.99, 118.86, 118.97, 121.56, 123.50, 123.72, 124.96, 127.62, 130.64, 135.51, 136.01, 136.73, 148.68, 160.53, 166.27, 198.83. HRMS calcd for $C_{24}H_{22}N_2O_5$ $[M+H]^+$: 418.1529, found: 418.1545.

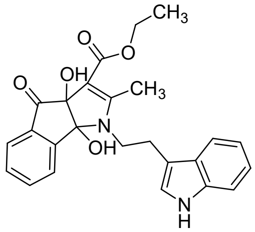

4b)***ethyl 1-(2-(1H-indol-3-yl)ethyl)-3a,8b-dihydroxy-2-methyl-4-oxo-1,3a,4,8b-tetrahydroindeno[1,2-b]pyrrole-3-carboxylate***

Yellow soild; m.p.: 121-123°C; IR(cm^{-1}): 744, 1175, 1339, 1414, 1551, 1655, 1717, 3408; ^1H NMR (400 MHz, DMSO-d_6; δ, ppm): 1.17 (t, *J*=8.0 Hz, 3H, CH_3), 2.19 (s, 3H, CH_3), 2.87-2.95 (m, 1H, CH), 3.09-3.17 (m, 1H, CH), 3.67-3.75 (m, 1H, CH), 3.91-4.06 (m, 3H, CH_2+CH), 5.55 (s, 1H, OH), 6.74 (s, 1H, OH), 6.99 (t, *J*=8.0 Hz, 1H, ArH), 7.06 (t, *J*=8.0 Hz, 1H, ArH), 7.26 (s, 1H, ArH), 7.33 (d, *J*=8.0 Hz, 1H, ArH), 7.53 (t, *J*=8.0 Hz, 1H, ArH), 7.59 (d, *J*=8.0 Hz, 1H, ArH), 7.67-7.74 (m, 2H, ArH), 7.86 (d, *J*=8.0 Hz, 1H, ArH), 10.89 (s, 1H, NH); ^{13}C NMR (100 MHz, DMSO-d_6; δ, ppm): 13.18, 15.13, 27.47, 43.08, 58.28, 84.98, 94.83, 95.10, 111.76, 111.98, 118.86, 118.96, 121.56, 123.49, 123.69, 124.97, 127.62, 130.63, 135.54, 135.98, 136.73, 148.73, 160.18, 166.79, 198.75. HRMS calcd for $C_{25}H_{24}N_2O_5$ $[M+H]^+$: 432.1685, found: 432.1701.

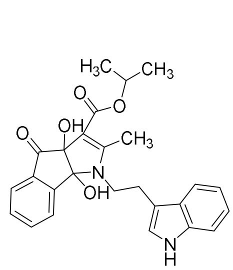

4c)***isopropyl 1-(2-(1H-indol-3-yl)ethyl)-3a,8b-dihydroxy-2-methyl-4-oxo-1,3a,4,8b-tetrahydroindeno[1,2-b]pyrrole-3-carboxylate***

Yellow soild; m.p.: 101-103°C; IR(cm^{-1}): 743, 1107, 1175, 1416, 1553, 1647, 1718, 3412; ^{1}H NMR (400 MHz, DMSO-d_6; δ, ppm): 1.18 (t, *J*=8.0 Hz, 6H, 2CH$_3$), 2.20 (s, 3H, CH$_3$), 2.86-2.94 (m, 1H, CH), 3.10-3.16 (m, 1H, CH), 3.67-3.75 (m, 1H, CH), 3.92-4.00 (m, 1H, CH), 4.81-4.87 (m, 1H, CH), 5.42 (s, 1H, OH), 6.72 (s, 1H, OH), 6.98 (t, *J*=8.0 Hz, 1H, ArH), 7.06 (t, *J*=8.0 Hz, 1H, ArH), 7.26 (s, 1H, ArH), 7.34 (d, *J*=8.0 Hz, 1H, ArH), 7.53 (t, *J*=8.0 Hz, 1H, ArH), 7.59 (d, *J*=8.0 Hz, 1H, ArH), 7.67-7.74 (m, 2H, ArH), 7.86 (d, *J*=8.0 Hz, 1H, ArH), 10.88 (s, 1H, NH); ^{13}C NMR (100 MHz, DMSO-d_6; δ, ppm): 13.22, 22.54, 27.50, 43.06, 65.28, 85.00, 95.04, 111.79, 111.98, 118.87, 118.96, 121.56, 123.50, 123.65, 124.98, 127.62, 130.62, 135.55, 135.96, 136.74, 148.83, 159.82, 165.35, 198.77. HRMS calcd for $C_{26}H_{26}N_2O_5$ [M+H]$^+$: 446.1842, found: 446.1838.

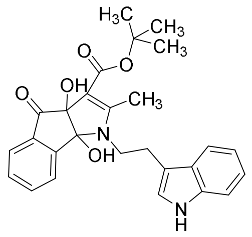

4d)***tert-butyl 1-(2-(1H-indol-3-yl)ethyl)-3a,8b-dihydroxy-2-methyl-4-oxo-1,3a,4,8b-tetrahydroi-ndeno[1,2-b]pyrrole-3-carboxylate***

Yellow soild; m.p.: 110-112°C; IR(cm^{-1}): 743, 1153, 1366, 1458, 1560, 1655, 1718, 3410; ^{1}H NMR (400 MHz, DMSO-d_6; δ, ppm): 1.41 (s, 9H, 3CH$_3$), 2.18 (s, 3H, CH$_3$), 2.85-2.92 (m, 1H, CH), 3.08-3.15 (m, 1H, CH), 3.66-3.74 (m, 1H, CH), 3.90-3.98 (m, 1H, CH), 5.28 (s, 1H, OH), 6.69 (s, 1H, OH), 6.99 (t, *J*=8.0 Hz, 1H, ArH), 7.06 (t, *J*=8.0 Hz, 1H, ArH), 7.25 (s, 1H, ArH), 7.34 (d, *J*=8.0 Hz, 1H, ArH), 7.53 (t, *J*=8.0 Hz, 1H, ArH), 7.59 (d, *J*=8.0 Hz, 1H, ArH), 7.68-7.74 (m, 2H, ArH), 7.85 (d, *J*=8.0 Hz, 1H, ArH), 10.88 (s, 1H, NH); ^{13}C NMR (100 MHz, DMSO-d_6; δ, ppm): 13.12, 27.53, 28.95, 43.01, 78.24, 84.97, 94.93, 96.01, 111.81, 111.98, 118.87, 118.96, 121.56, 123.50, 123.62, 124.96, 127.62, 130.60, 135.54, 135.95, 136.74, 148.96, 159.31, 165.47, 198.94. HRMS calcd for $C_{27}H_{28}N_2O_5$ [M+H]$^+$: 460.1998, found: 460.1969.

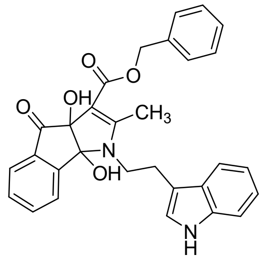

4e)***benzyl 1-(2-(1H-indol-3-yl)ethyl)-3a,8b-dihydroxy-2-methyl-4-oxo-1,3a,4,8b-tetrahydroindeno[1,2-b]pyrrole-3-carboxylate***

Yellow soild; m.p.: 100-102°C; IR(cm^{-1}): 743, 1157, 1340, 1412, 1547, 1655, 1718, 3423; ^{1}H NMR (400 MHz, DMSO-d_6; δ, ppm): 2.21 (s, 3H, CH$_3$), 2.89-2.96 (m, 1H, CH), 3.11-3.18 (m, 1H, CH), 3.70-3.78 (m, 1H, CH), 3.96-4.03 (m, 1H, CH), 5.28 (s, 1H, OH), 6.69 (s, 1H, OH), 5.08 (s, 2H, CH$_2$), 5.69 (s, 1H, OH), 6.80 (s, 1H, OH), 6.99 (t, *J*=8.0 Hz, 1H, ArH), 7.06 (t, *J*=8.0 Hz, 1H,

ArH), 7.22-7.26 (m, 2H, ArH),7.31-7.35 (m, 3H, ArH), 7.46 (d, *J*=8.0 Hz, 1H, ArH), 7.54 (t, *J*=8.0 Hz, 1H, ArH), 7.60 (d, *J*=8.0 Hz, 1H, ArH), 7.69-7.75 (m, 2H, ArH), 7.87 (d, *J*=8.0 Hz, 1H, ArH), 10.89 (s, 1H, NH); ^{13}C NMR (100 MHz, DMSO-d_6; δ, ppm): 13.33, 27.42, 43.16, 63.97, 85.10, 94.57, 95.20, 111.74, 111.99, 118.87, 118.98, 121.57, 123.54, 123.71, 125.02, 127.63, 127.69, 128.65, 130.69, 135.60, 136.04, 136.74, 138.43, 148.66, 160.99, 166.58, 198.88.; HRMS calcd for $C_{30}H_{26}N_2O_5$ [M+H]$^+$: 494.1842, found: 494.1853.

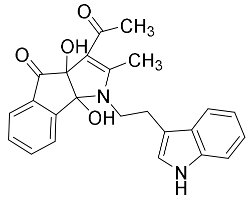

4f)***1-(2-(1H-indol-3-yl)ethyl)-3-acetyl-3a,8b-dihydroxy-2-methyl-3a,8b-dihydroindeno[1,2-b]pyrrol-4(1H)-one***

Yellow soild; m.p.: 164-166°C; IR(cm^{-1}): 748, 1356, 1406, 1481, 1601, 1718, 3412; ^{1}H NMR (400 MHz, DMSO-d_6; δ, ppm): 2.26 (s, 3H, CH_3), 2.31 (s, 3H, CH_3), 2.85-2.89(m, 1H, CH), 3.10-3.15 (m, 1H, CH), 3.71-3.76 (m, 1H, CH), 3.91-3.96 (m, 1H, CH), 6.02 (s, 1H, OH), 6.89 (s, 1H, OH), 6.99 (t, *J*=8.0 Hz, 1H, ArH), 7.06 (t, *J*=8.0 Hz, 1H, ArH), 7.26 (s, 1H, ArH), 7.34 (d, *J*=8.0 Hz, 1H, ArH), 7.57 (t, *J*=8.0 Hz, 2H, ArH), 7.72-7.79 (m, 2H, ArH), 7.91 (d, *J*=8.0 Hz, 1H, ArH), 10.88 (s, 1H, NH); ^{13}C NMR (100 MHz, DMSO-d_6; δ, ppm): 13.92, 27.20, 30.08, 42.83, 85.36, 95.25, 106.97, 111.67, 112.02, 118.79, 119.02, 121.61, 123.65, 123.95, 125.16, 127.57, 130.77, 135.25, 136.45, 136.73, 149.34, 160.59, 193.57, 200.43.; HRMS calcd for $C_{24}H_{22}N_2O_4$ [M+H]$^+$: 402.7580, found: 402.1596.

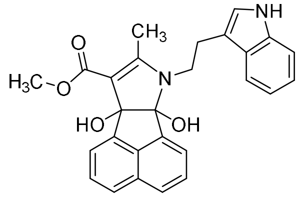

6a)***methyl 7-(2-(1H-indol-3-yl)ethyl)-6b,9a-dihydroxy-8-methyl-6b,9a-dihydro-7H-acenaphtho-[1,2-b]pyrrole-9-carboxylate***

White soild; m.p.: 161-163°C; IR(cm^{-1}): 746, 787, 833, 1003, 1080, 1198, 1383, 1439, 1560, 1637, 3421; ^{1}H NMR (400 MHz, DMSO-d_6; δ, ppm): 2.19 (s, 3H, CH_3), 2.84-2.91 (m, 1H, CH), 3.06-3.13 (m, 1H, CH), 3.66 (s, 3H, CH_3), 3.70-3.76 (m, 2H, CH_2), 5.51 (s, 1H, OH), 6.49 (s, 1H, OH), 6.99 (t, *J*=8.0 Hz, 1H, ArH), 7.07 (t, *J*=8.0 Hz, 1H, ArH), 7.25 (s, 1H, ArH), 7.34 (d, *J*=8.0 Hz, 1H, ArH), 7.52-7.62 (m, 4H, ArH), 7.70 (d, *J*=8.0 Hz, 1H, ArH), 7.76-7.82 (m, 2H, ArH), 10.90 (s, 1H, NH); ^{13}C NMR (100 MHz, DMSO-d_6; δ, ppm): 13.05, 19.11, 27.00, 43.19, 50.08, 56.58, 87.65, 99.60, 100.90, 111.96, 112.03, 118.75, 118.86, 119.60, 121.57, 123.58, 123.85, 125.27, 127.62, 128.27, 129.10, 131.30, 136.45, 136.75, 142.28, 145.98, 160.48, 166.46. HRMS calcd for $C_{27}H_{24}N_2O_4$ [M+H]$^+$: 440.1736, found: 440.1745.

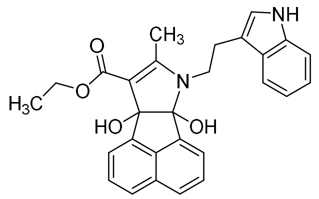

6b)***ethyl 7-(2-(1H-indol-3-yl)ethyl)-6b,9a-dihydroxy-8-methyl-6b,9a-dihydro-7H-acenaphtho[1, 2-b] pyrrole-9-carboxylate***

White soild; m.p.: 145-147°C; IR(cm^{-1}): 748, 785, 833, 1080, 1173, 1188, 1339, 1418, 1566, 1618, 3366; ^{1}H NMR (400 MHz, DMSO-*d$_6$*; δ, ppm): 1.28 (t, *J*=8.0 Hz, 3H, CH_3), 2.20 (s, 3H, CH_3), 2.83-2.90 (m, 1H, CH), 3.05-3.12 (m, 1H, CH), 3.67-3.78 (m, 2H, CH_2), 4.07-4.19 (m, 2H, CH_2), 5.47 (s, 1H, OH), 6.46 (s, 1H, OH), 6.99 (t, *J*=8.0 Hz, 1H, ArH), 7.06 (t, *J*=8.0 Hz, 1H, ArH), 7.25 (s, 1H, ArH), 7.34 (d, *J*=8.0 Hz, 1H, ArH), 7.52-7.61 (m, 4H, ArH), 7.70 (d, *J*=8.0 Hz, 1H, ArH), 7.77 (d, *J*=8.0 Hz, 1H, ArH), 7.85 (d, *J*=8.0 Hz, 1H, ArH), 10.89 (s, 1H, NH); ^{13}C NMR (100 MHz, DMSO-*d$_6$*; δ, ppm): 13.13, 15.38, 27.01, 43.18, 58.28, 87.67, 99.72, 100.84, 111.96, 112.02, 118.75, 118.97, 119.56, 121.55, 121.71, 123.56, 123.81, 125.24, 127.61, 128.26, 129.03, 131.28, 136.43, 136.73, 142.29, 145.96, 160.24, 166.02. HRMS calcd for $C_{28}H_{26}N_2O_4$ $[M+H]^+$: 454.1893, found: 454.1931.

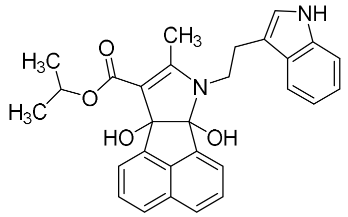

6c) ***isopropyl 7-(2-(1H-indol-3-yl)ethyl)-6b,9a-dihydroxy-8-methyl-6b,9a-dihydro-7H-acenaphtho [1,2-b]pyrrole-9-carboxylate***

White soild; m.p.: 161-163°C; IR(cm^{-1}): 731, 785, 833, 997, 1080, 1107, 1175, 1400, 1568, 1605, 3325; ^{1}H NMR (400 MHz, DMSO-*d$_6$*; δ, ppm): 1.27 (dd, J_1=8.0 Hz, J_2=16.0 Hz, 6H, $2CH_3$), 2.21 (s, 3H, CH_3), 2.81-2.89 (m, 1H, CH), 3.04-3.11 (m, 1H, CH), 3.69-3.74 (m, 2H, CH_2), 4.97-5.03 (m, 1H, CH), 5.42 (s, 1H, OH), 6.45 (s, 1H, OH), 6.99 (t, *J*=8.0 Hz, 1H, ArH), 7.06 (t, *J*=8.0 Hz, 1H, ArH), 7.25 (s, 1H, ArH), 7.34 (d, *J*=8.0 Hz, 1H, ArH), 7.52-7.61 (m, 4H, ArH), 7.70 (d, *J*=8.0 Hz, 1H, ArH), 7.77 (d, *J*=8.0 Hz, 1H, ArH), 7.90 (d, *J*=8.0 Hz, 1H, ArH), 10.89 (s, 1H, NH); ^{13}C NMR (100 MHz, DMSO-*d$_6$*; δ, ppm): 13.22, 22.83, 22.91, 27.04, 43.17, 65.10, 87.69, 99.96, 100.80, 111.97, 112.02, 118.75, 118.97, 119.52, 121.55, 121.77, 123.53, 123.80, 125.21, 127.60, 128.24, 128.99, 131.27, 136.43, 136.74, 142.29, 145.98, 159.94, 165.57. HRMS calcd for $C_{29}H_{28}N_2O_4$ $[M+H]^+$: 468.2049, found: 468.2066.

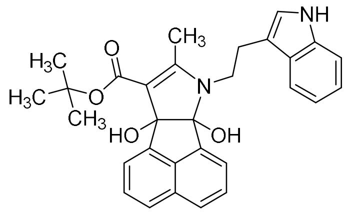

6d) ***tert-butyl 7-(2-(1H-indol-3-yl)ethyl)-6b,9a-dihydroxy-8-methyl-6b,9a-dihydro-7H-acenap htho[1,2-b]pyrrole-9-carboxylate***

White soild; m.p.: 112-114°C; IR(cm^{-1}): 741, 785, 833, 1080, 1150, 1366, 1393, 1572, 1649, 3349; ^{1}H NMR (400 MHz, DMSO-d_6; δ, ppm): 1.50 (s, 9H, 3CH_3), 2.18 (s, 3H, CH_3), 2.81 (s, 1H, CH), 3.05 (s, 1H, CH), 3.68 (s, 2H, CH_2), 5.34 (s, 1H, OH), 6.42 (s, 1H, OH), 6.99 (t, J=8.0 Hz, 1H, ArH), 7.06 (t, J=8.0 Hz, 1H, ArH), 7.24 (s, 1H, ArH), 7.34 (d, J=8.0 Hz, 1H, ArH), 7.51-7.60 (m, 4H, ArH), 7.70 (d, J=8.0 Hz, 1H, ArH), 7.77 (d, J=8.0 Hz, 1H, ArH), 7.91 (d, J=8.0 Hz, 1H, ArH), 10.89 (s, 1H, NH); ^{13}C NMR (100 MHz, DMSO-d_6; δ, ppm): 13.24, 19.10, 27.07, 29.19, 43.11, 56.56, 78.11, 87.72, 100.68, 100.99, 112.00, 118.74, 118.96, 119.45, 121.41, 121.54, 123.49, 123.78, 125.15, 127.59, 128.24, 129.07, 131.29, 136.41, 136.73, 142.34, 146.23, 159.12, 165.79. HRMS calcd for $C_{30}H_{30}N_2O_4$ [M+H]$^+$: 482.2206, found: 482.2245.

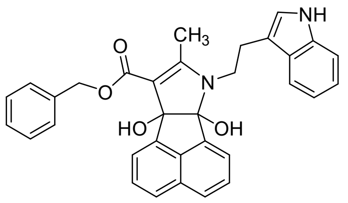

6e) ***benzyl 7-(2-(1H-indol-3-yl)ethyl)-6b,9a-dihydroxy-8-methyl-6b,9a-dihydro-7H-acenaphtho [1,2-b]pyrrole-9-carboxylate***

White soild; m.p.: 95-97°C; IR(cm^{-1}): 699, 743, 783, 833, 1080, 1167, 1190, 1346, 1416, 1456, 1560, 1649, 3416; ^{1}H NMR (400 MHz, DMSO-d_6; δ, ppm): 2.22 (s, 3H, CH_3), 2.87 (s, 1H, CH), 3.07 (s, 1H, CH), 3.75 (s, 2H, CH_2), 5.21 (s, 2H, CH_2), 5.56 (s, 1H, OH), 6.52 (s, 1H, OH), 6.99 (t, J=8.0 Hz, 1H, ArH), 7.06 (t, J=8.0 Hz, 1H, ArH), 7.26-7.36 (m, 5H, ArH), 7.41-7.47 (m, 3H, ArH), 7.54-7.62 (m, 3H, ArH), 7.68 (d, J=8.0 Hz, 1H, ArH), 7.78 (d, J=8.0 Hz, 2H, ArH), 10.90 (s, 1H, NH); ^{13}C NMR (100 MHz, DMSO-d_6; δ, ppm): 13.22, 26.97, 43.26, 63.98, 87.73, 99.42, 100.92, 111.95, 112.02, 118.75, 118.98, 119.62, 121.55, 121.87, 123.56, 123.84, 125.29, 127.61, 127.97, 128.22, 128.83, 128.95, 131.27, 136.43, 136.75, 138.47, 142.19, 145.83, 160.85, 165.73. HRMS calcd for $C_{33}H_{28}N_2O_4$ [M+H]$^+$: 516.2049, found: 516.2058.

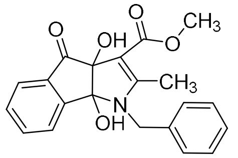

8a)***methyl 1-benzyl-3a,8b-dihydroxy-2-methyl-4-oxo-1,3a,4,8b-tetrahydroindeno[1,2-b]pyrrole-3-carboxylate***

White soild; m.p.: 154-156°C; IR(cm^{-1}): 784, 823, 1101, 1296, 1427, 1562, 1635, 1721, 3398; ^{1}H NMR (400 MHz, DMSO-d_6; δ, ppm): 1.93 (s, 3H, CH_3), 3.50 (s, 3H, CH_3), 4.75 (d, J=16.0 Hz, 1H, CH), 5.12 (d, J=16.0 Hz, 1H, CH), 5.70 (s, 1H, OH), 6.79 (s, 1H, OH), 7.19-7.29 (m, 5H, ArH), 7.53 (t, J=8.0 Hz, 1H, ArH), 7.65-7.70(m, 2H, ArH), 7.77 (d, J=8.0Hz, 1H, ArH); ^{13}C NMR (100 MHz, DMSO-d_6; δ, ppm): 13.65, 45.38, 50.20, 85.04, 94.63, 95.59, 123.46, 125.27, 127.23, 127.37, 128.84, 130.65, 135.49, 135.86, 139.40, 148.54, 160.86, 166.19, 198.81. HRMS calcd for $C_{21}H_{19}NO_5$ [M+H]$^+$: 365.1263, found: 365.1279.

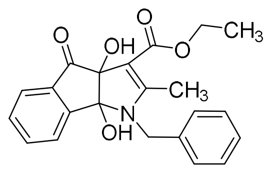

8b)***ethyl 1-benzyl-3a,8b-dihydroxy-2-methyl-4-oxo-1,3a,4,8b-tetrahydroindeno[1,2-b]pyrrole-3-carboxylate***

Yellow soild; m.p.: 72-73°C; IR(cm^{-1}): 765, 852, 1091, 1216, 1437, 1545, 1629, 1743, 3421; ^{1}H NMR (400 MHz, DMSO-d_6; δ, ppm): 1.15 (t, J=8.0 Hz, 3H, CH_3), 1.92 (s, 3H, CH_3), 3.90-4.05 (m, 2H, CH_2), 4.75 (d, J=16.0 Hz, 1H, CH), 5.10 (d, J=16.0 Hz, 1H, CH), 5.61 (s, 1H, OH), 6.78 (s, 1H, OH), 7.20-7.29 (m, 5H, ArH), 7.53 (t, J=8.0 Hz, 1H, ArH), 7.64-7.70 (m, 2H, ArH), 7.76 (d, J=8.0 Hz, 1H, ArH); ^{13}C NMR (100 MHz, DMSO-d_6; δ, ppm): 13.67, 15.08, 45.36, 58.37, 85.07, 94.58, 95.67, 123.45, 125.27, 127.22, 127.36, 128.83, 130.64, 135.52, 135.81, 139.44, 148.56, 160.50, 165.70, 198.73. HRMS calcd for $C_{22}H_{21}NO_5$ $[M+H]^+$: 379.1420, found: 379.1438.

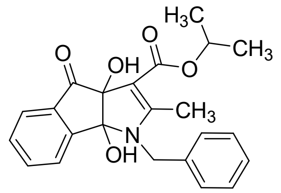

8c)***isopropyl 1-benzyl-3a,8b-dihydroxy-2-methyl-4-oxo-1,3a,4,8b-tetrahydroindeno[1,2-b]pyrrole-3-carboxylate***

Pale yellow soild; m.p.: 71-73°C; IR(cm^{-1}): 756, 918, 1096, 1285, 1408, 1533, 1667, 1715, 3426; ^{1}H NMR (400 MHz, DMSO-d_6; δ, ppm): 1.15 (t, J=8.0 Hz, 6H, $2CH_3$), 1.92 (s, 3H, CH_3), 4.73-4.84 (m, 2H, CH_2), 5.08 (d, J=16.0 Hz, 1H, CH), 5.50 (s, 1H, OH), 6.77 (s, 1H, OH), 7.19-7.28 (m, 5H, ArH), 7.52 (t, J=8.0 Hz, 1H, ArH), 7.64-7.70 (m, 2H, ArH), 7.76 (d, J=8.0 Hz, 1H, ArH); ^{13}C NMR (100 MHz, DMSO-d_6; δ, ppm): 13.69, 22.59, 45.33, 65.40, 85.08, 94.54, 95.85, 123.44, 125.28, 127.23, 127.35, 128.83, 130.63, 135.54, 135.79, 139.46, 148.62, 160.16, 165.25, 198.74. HRMS calcd for $C_{23}H_{23}NO_5$ $[M+H]^+$: 393.1576, found: 393.1561.

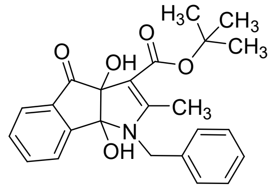

8d)***tert-butyl 1-benzyl-3a,8b-dihydroxy-2-methyl-4-oxo-1,3a,4,8b-tetrahydroindeno[1,2-b]pyrrole-3-carboxylate***

Yellow soild; m.p.: 83-85°C; IR(cm^{-1}): 749, 925, 1163, 1276, 1418, 1556, 1638, 1729, 3398; ^{1}H NMR (400 MHz, DMSO-d_6; δ, ppm): 1.38 (s, 9H, $3CH_3$), 1.89 (s, 3H, CH_3), 4.74 (d, J=16.0 Hz, 1H, CH), 5.06 (d, J=16.0 Hz, 1H, CH), 5.37 (s, 1H, OH), 6.74 (s, 1H, OH), 7.18-7.28 (m, 5H, ArH), 7.52 (t, J=8.0 Hz, 1H, ArH), 7.65 (t, J=8.0 Hz, 1H, ArH), 7.70 (d, J=8.0 Hz, 1H, ArH), 7.72 (d, J=8.0 Hz, 1H, ArH); ^{13}C NMR (100 MHz, DMSO-d_6; δ, ppm): 13.58, 28.89, 45.28, 78.36, 85.06, 94.46, 96.84, 123.44, 125.26, 127.21, 127.33, 128.82, 130.61, 135.54, 135.77, 139.53, 148.72, 159.64, 165.33, 198.92. HRMS calcd for $C_{24}H_{25}NO_5$ $[M+H]^+$: 407.1733, found: 407.1749.

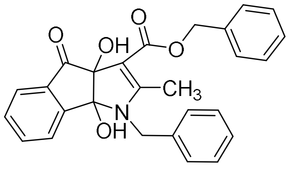

8e)***benzyl 1-benzyl-3a,8b-dihydroxy-2-methyl-4-oxo-1,3a,4,8b-tetrahydroindeno[1,2-b]pyrrole-3-carboxylate***

Yellow soild; m.p.: 70-71°C; IR(cm^{-1}): 732, 838, 1119, 1239, 1430, 1562, 1669, 1728, 3411; ^{1}H NMR (400 MHz, DMSO-*d$_6$*; δ, ppm): 1.94 (s, 3H, CH_3), 4.77 (d, *J*=16.0 Hz, 1H, CH), 5.02-5.14 (m, 3H, CH_2+CH), 5.75 (s, 1H, OH), 6.84 (s, 1H, OH), 7.20-7.33 (m, 8H, ArH), 7.45 (d, *J*=8.0 Hz, 1H, ArH), 7.54 (t, *J*=8.0 Hz, 1H, ArH), 7.65-7.73 (m, 2H, ArH), 7.77 (d, *J*=8.0 Hz, 1H, ArH); ^{13}C NMR (100 MHz, DMSO-*d$_6$*; δ, ppm): 13.81, 45.53, 64.07, 85.16, 94.69, 95.40, 123.50, 125.33, 127.24, 127.38, 127.71, 127.76, 128.64, 128.85, 130.70, 135.58, 135.87, 138.29, 139.31, 148.50, 161.33, 166.52, 198.87. HRMS calcd for $C_{27}H_{23}NO_5$ $[M+H]^+$: 441.1576, found: 441.1589.

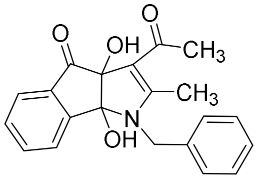

8f)***3-acetyl-1-benzyl-3a,8b-dihydroxy-2-methyl-3a,8b-dihydroindeno[1,2-b]pyrrol-4(1H)-one***

White soild; m.p.: 175-177°C; IR(cm^{-1}): 756, 841, 1093, 1216, 1435, 1562, 1674, 1735, 3417; ^{1}H NMR (400 MHz, DMSO-*d$_6$*; δ, ppm): 1.97 (s, 3H, CH_3), 2.31 (s, 3H, CH_3), 4.81 (d, *J*=16.0 Hz, 1H, CH), 5.05 (d, *J*=16.0 Hz, 1H, CH), 6.05 (s, 1H, OH), 6.96 (s, 1H, OH), 7.11 (d, *J*=8.0 Hz, 2H, ArH), 7.16-7.25(m, 3H, ArH), 7.54 (t, *J*=8.0 Hz, 1H, ArH), 7.65 (t, *J*=8.0 Hz, 1H, ArH), 7.73-7.79 (m, 2H, ArH); ^{13}C NMR (100 MHz, DMSO-*d$_6$*; δ, ppm): 14.32, 30.07, 45.21, 85.42, 94.79, 106.51, 123.88, 125.53, 127.05, 127.32, 128.81, 130.73, 135.23, 136.23, 139.04, 149.25, 160.80, 193.76, 200.46. HRMS calcd for $C_{21}H_{19}NO_4$ $[M+H]^+$: 349.1314, found: 349.1325.

4. Original ^{1}HNMR and ^{13}CNMR spectra

[1]H for 4a

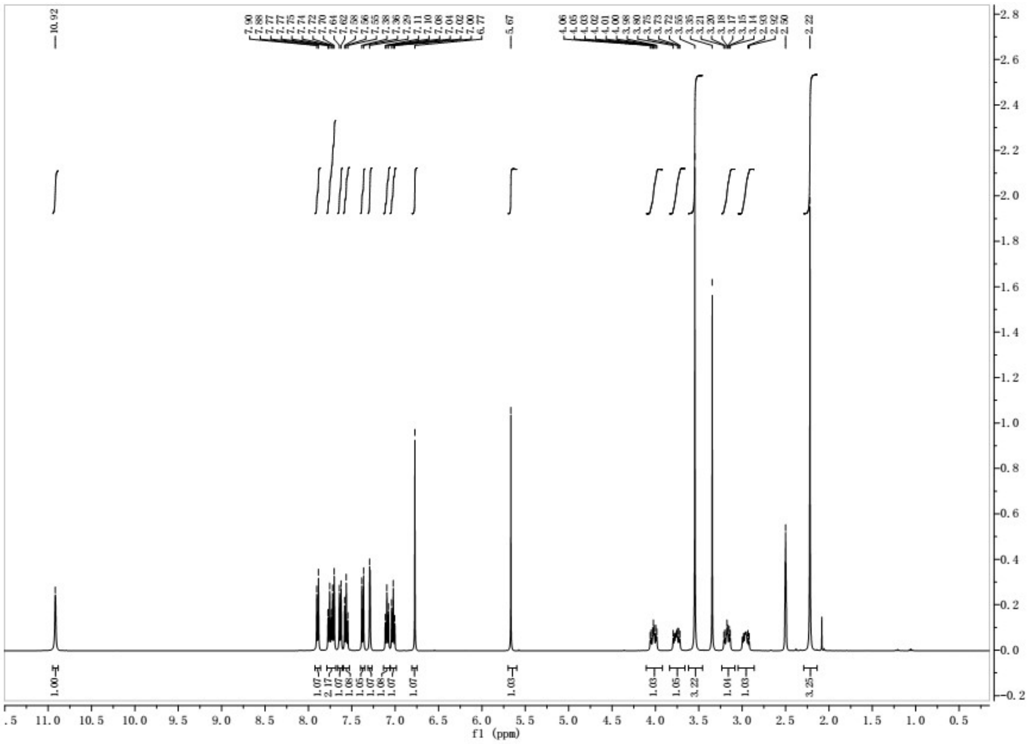

[13]C for 4a

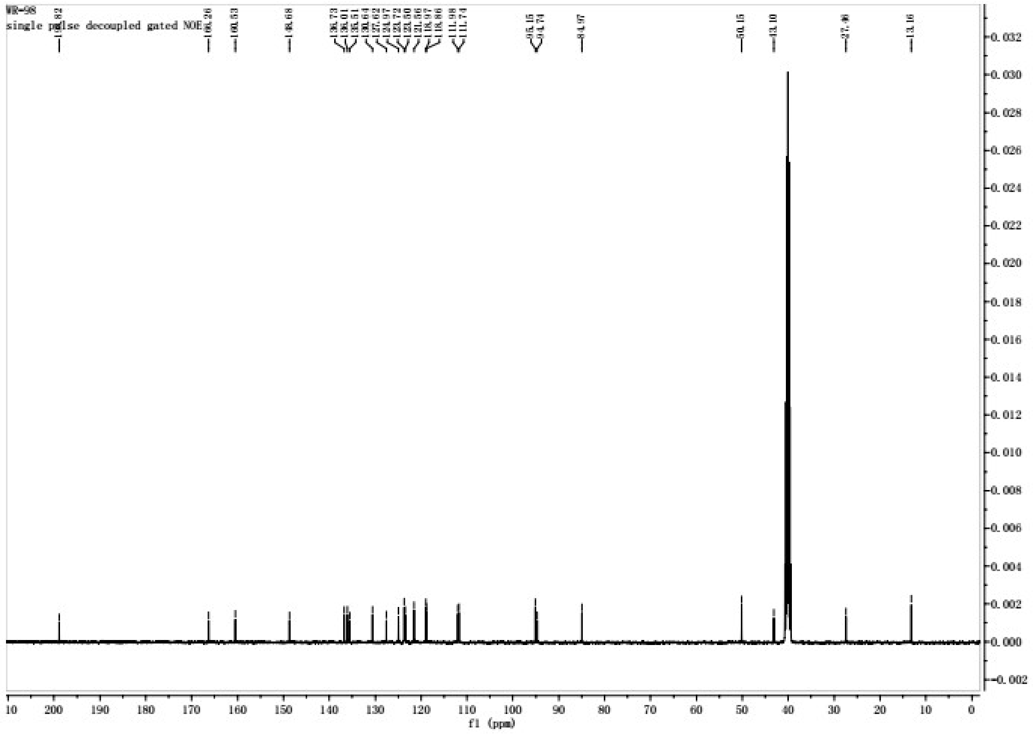

^{1}H for 4b

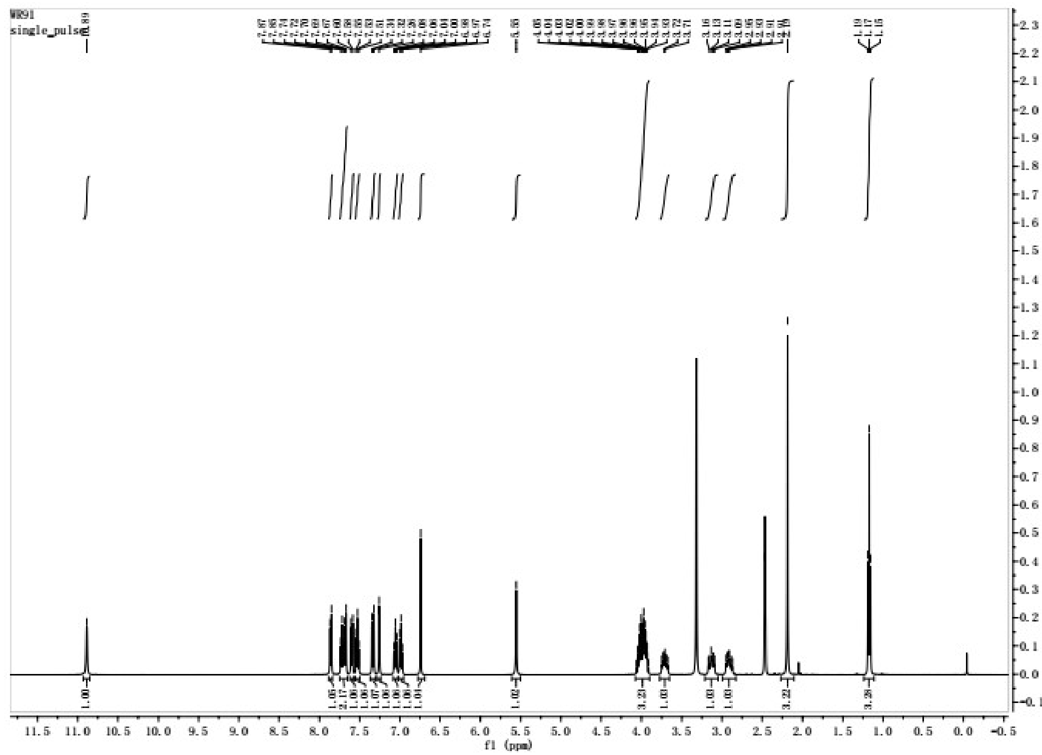

^{13}C for 4b

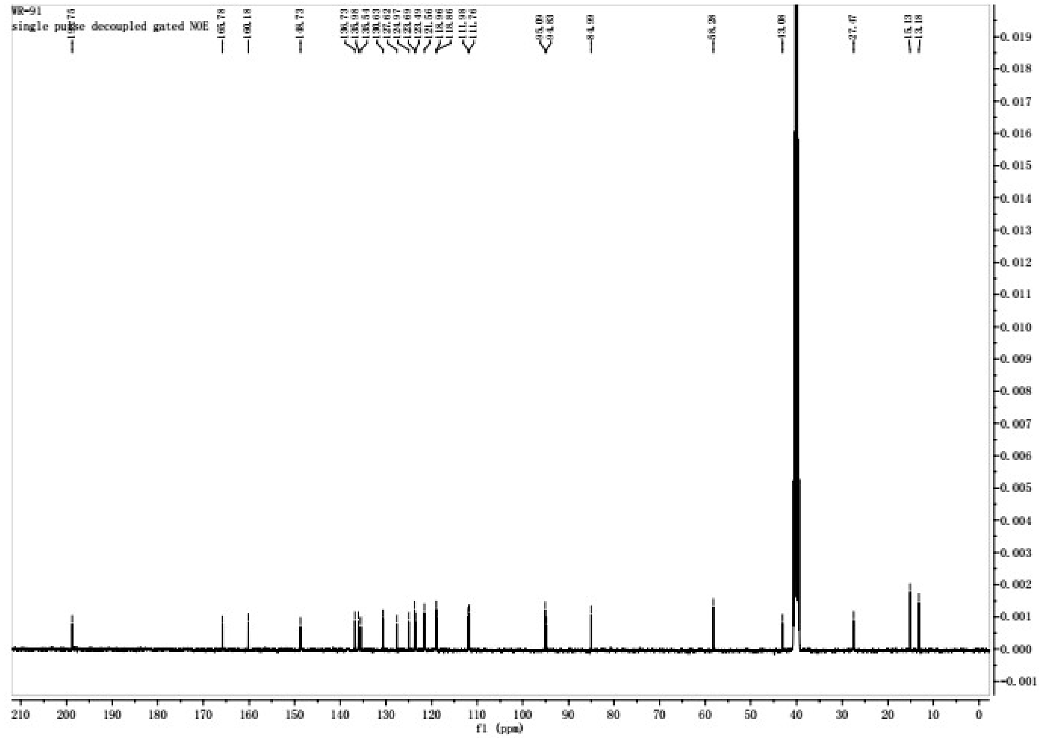

^{1}H for 4c

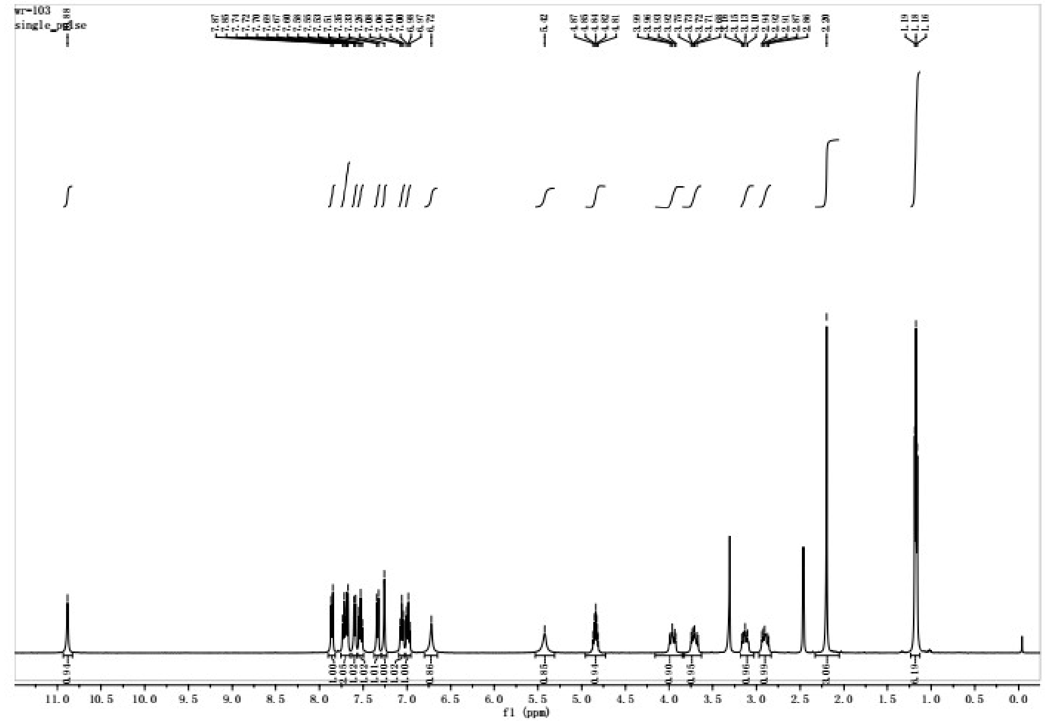

^{13}C for 4c

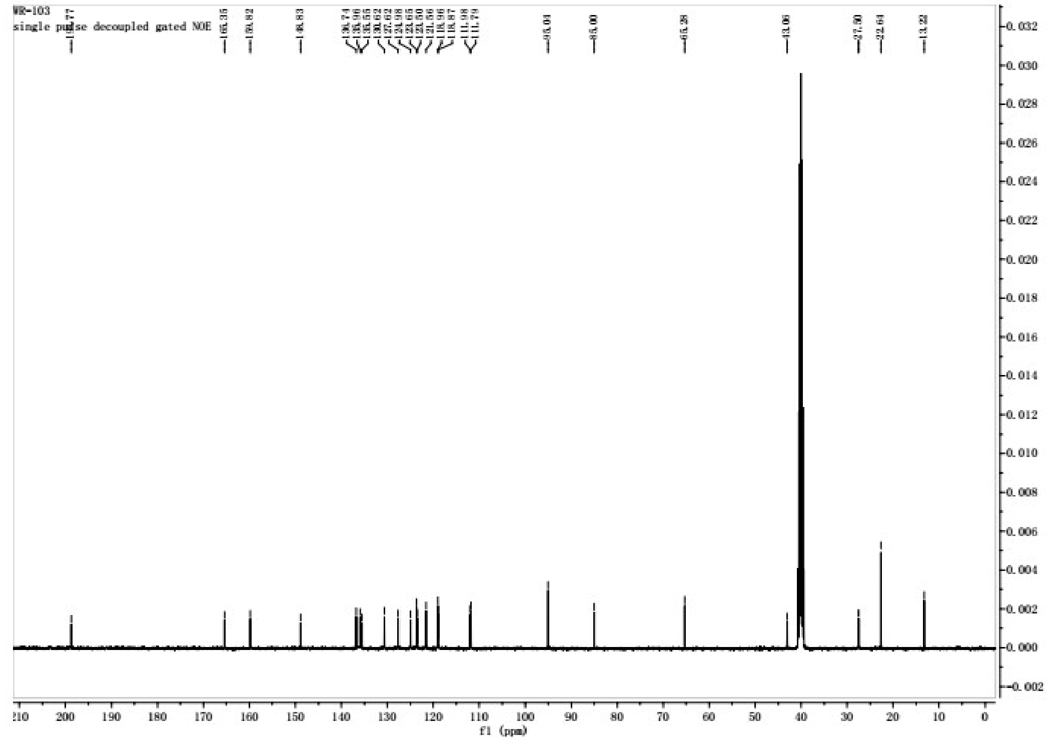

^{1}H for 4d

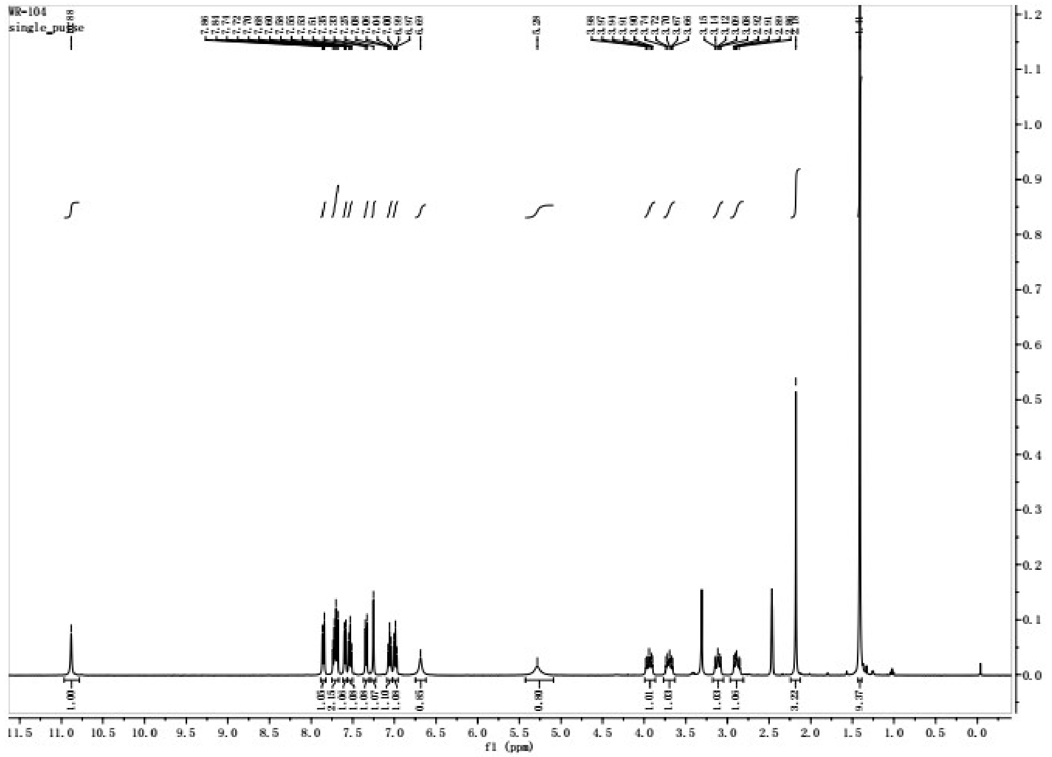

^{13}C for 4d

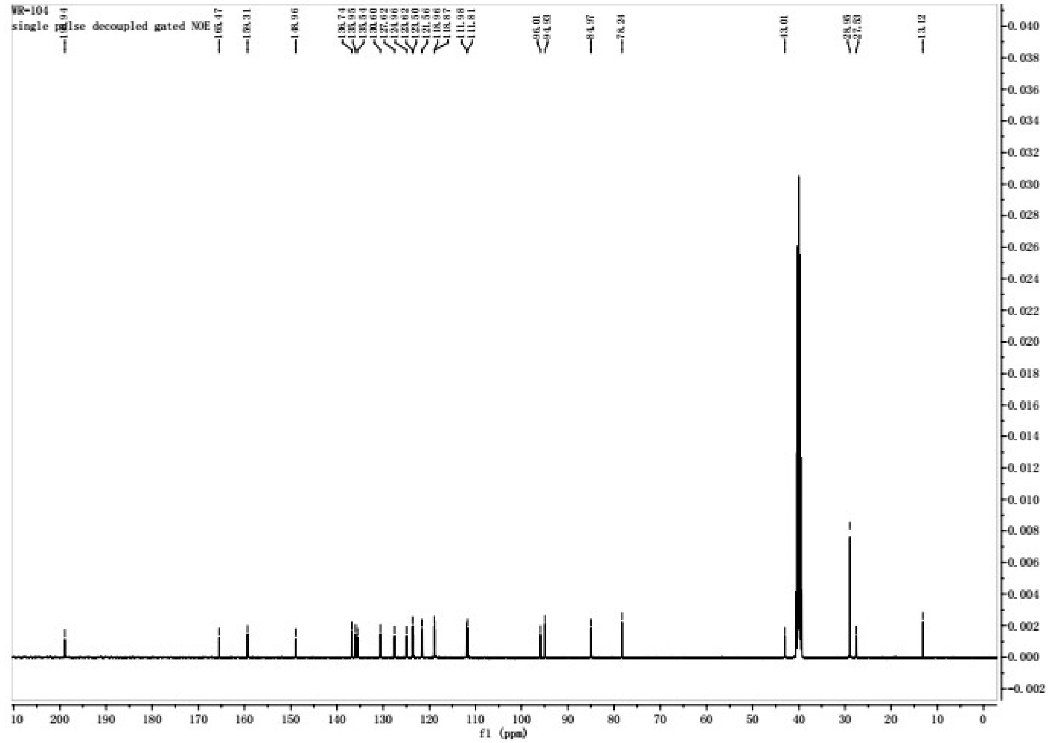

[1]H for 4e

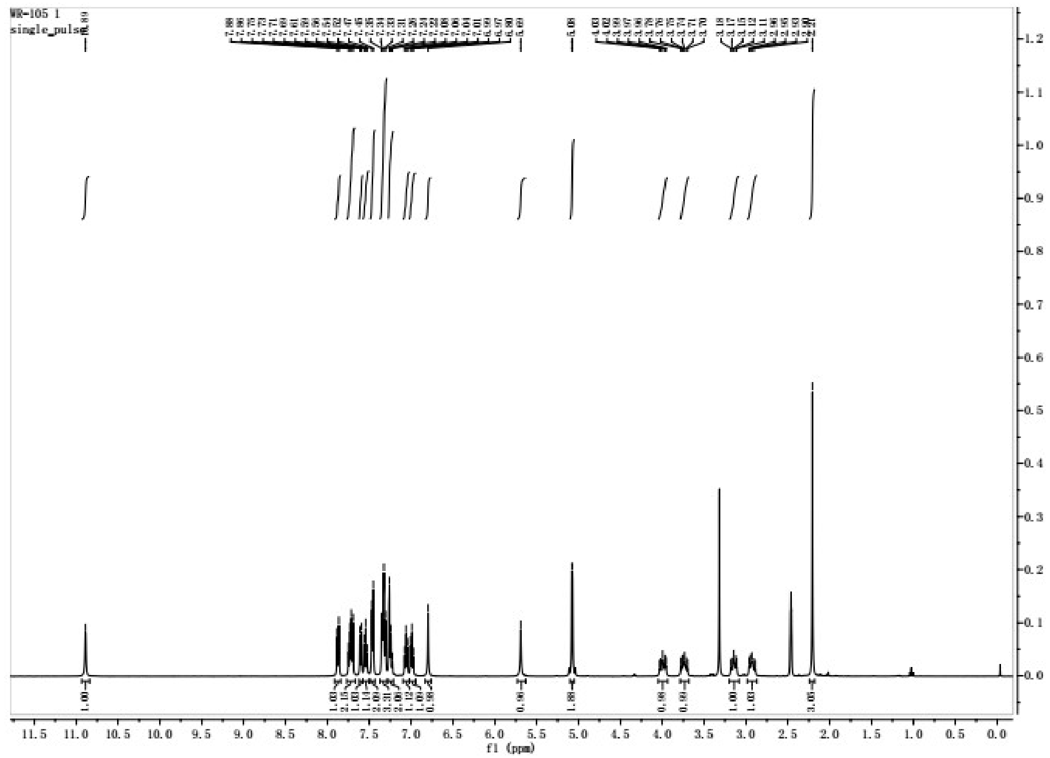

[13]C for 4e

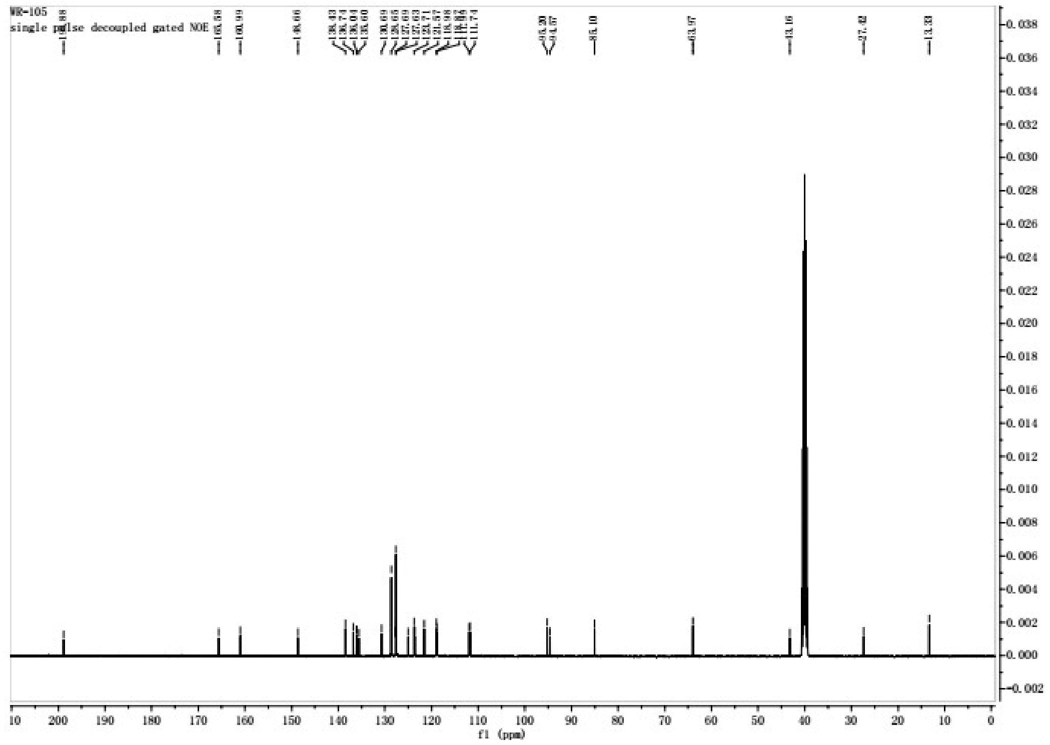

^{1}H for 4f

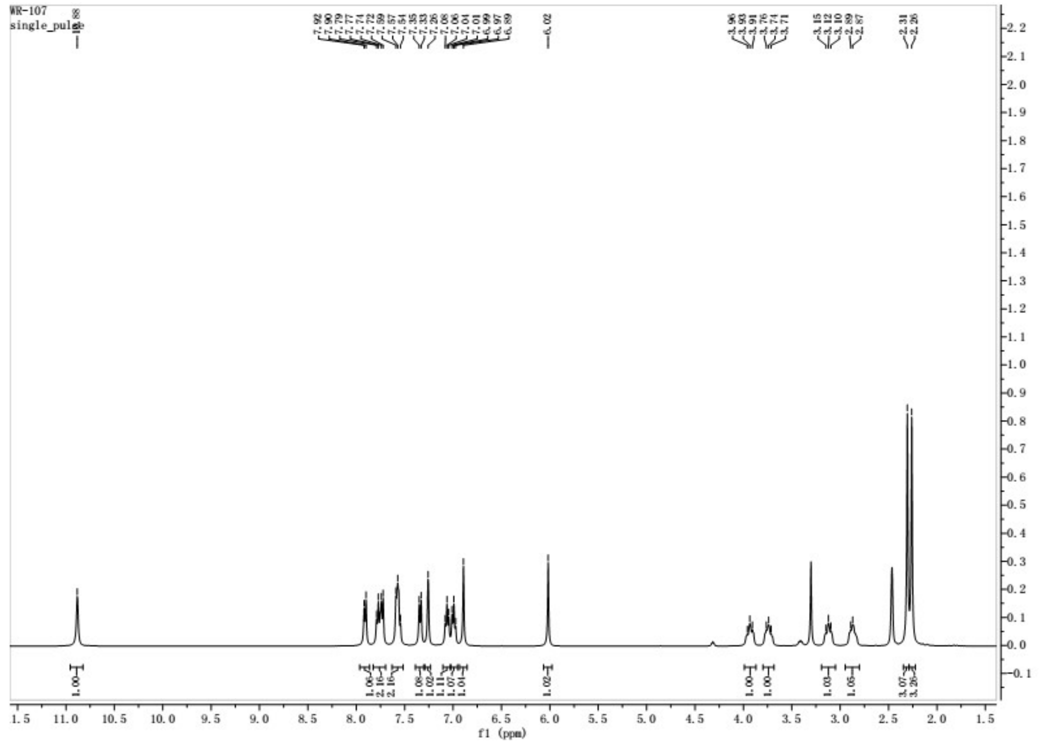

^{13}C for 4f

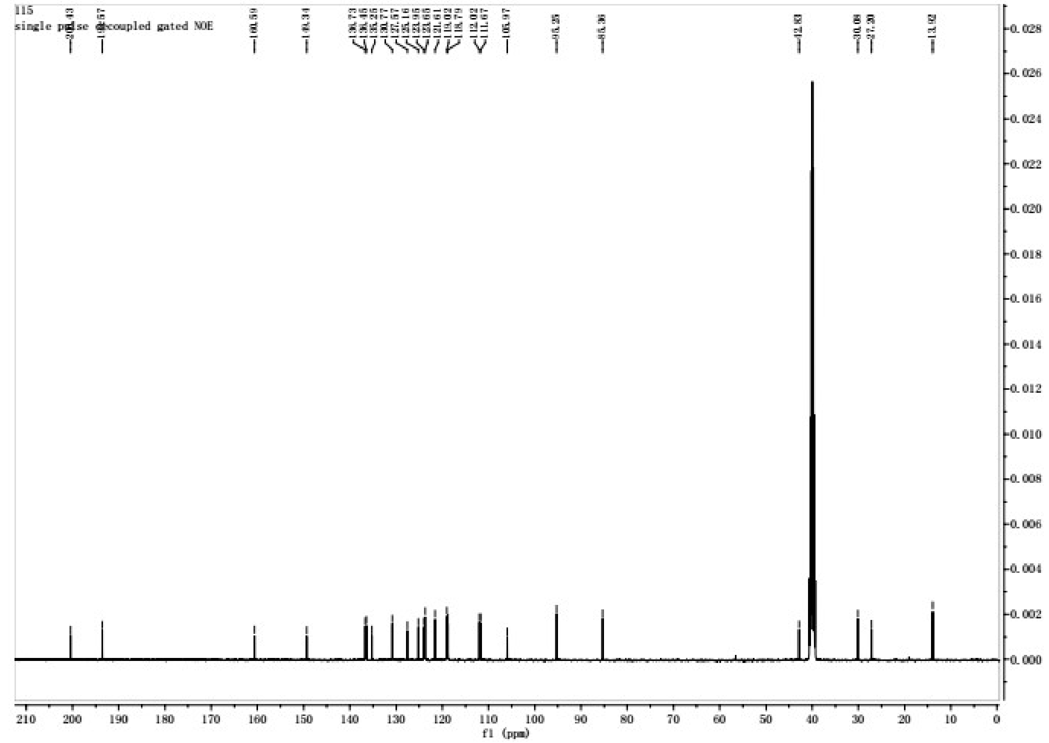

^{1}H for 6a

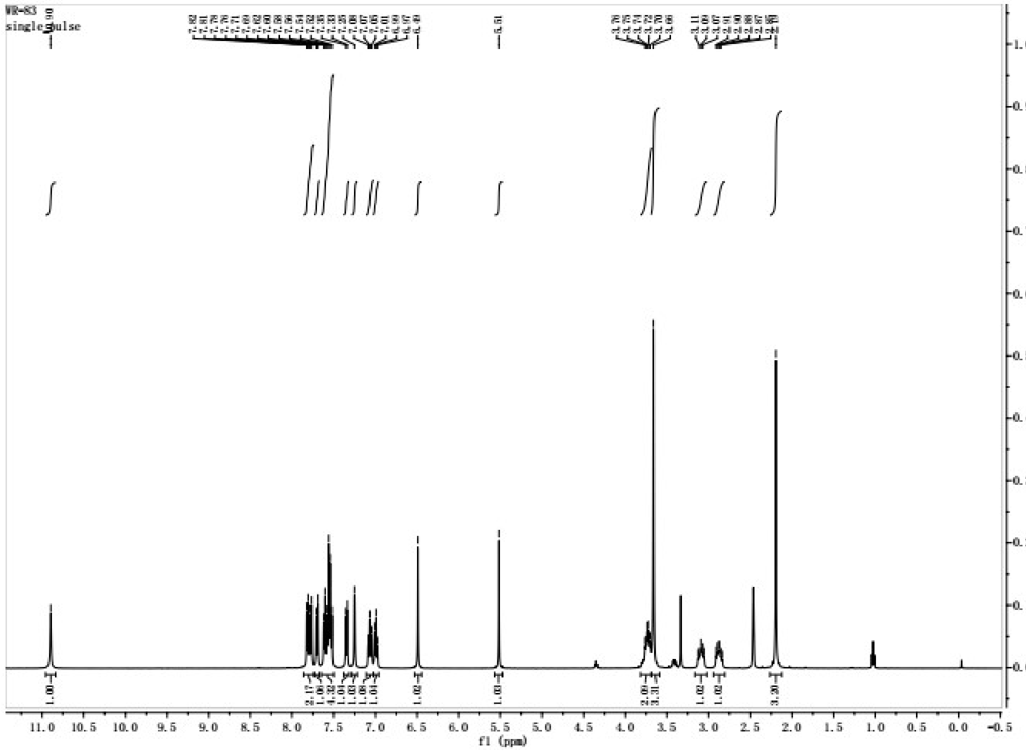

^{13}C for 6a

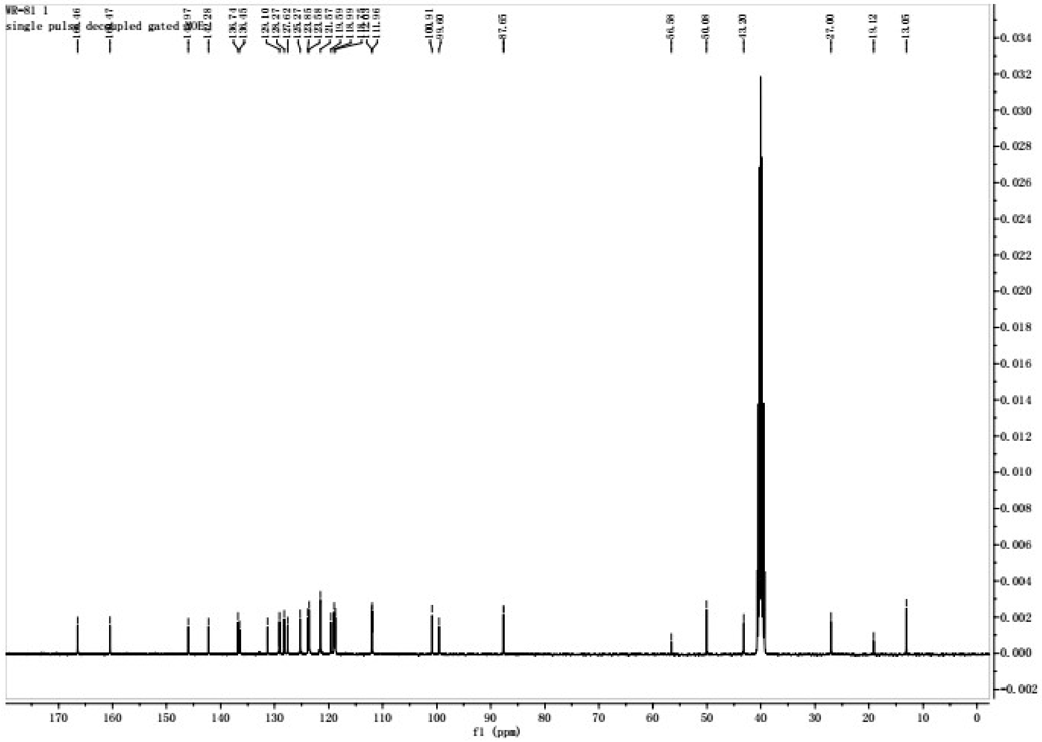

^{1}H for 6b

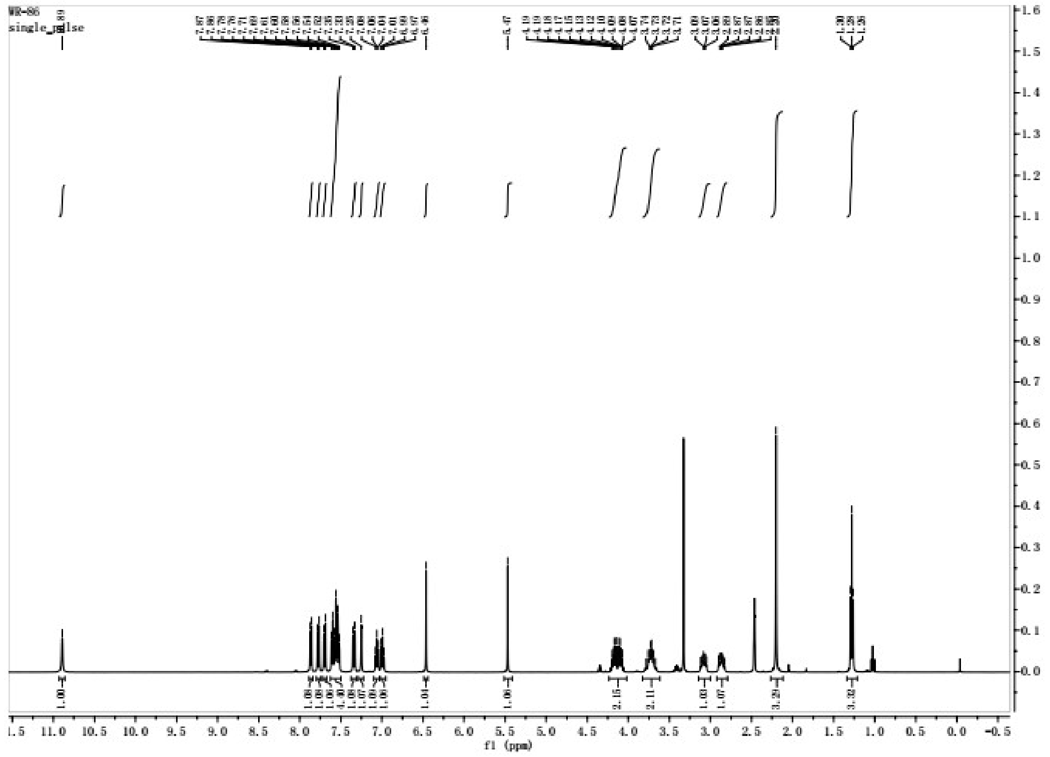

^{13}C for 6b

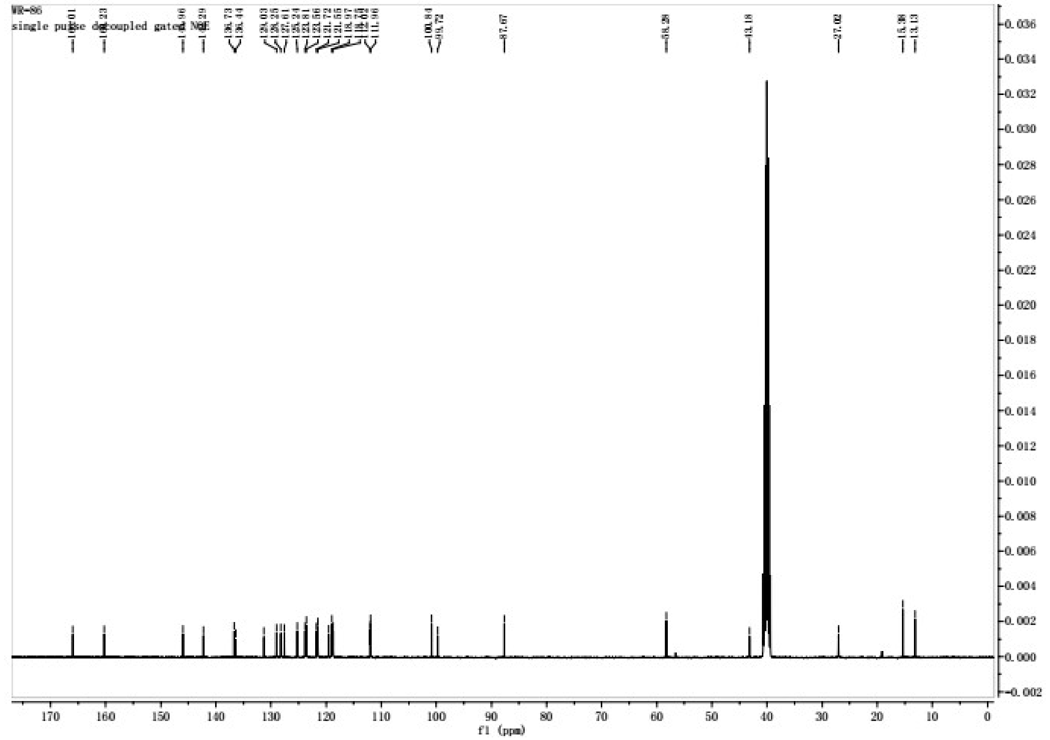

[1]H for 6c

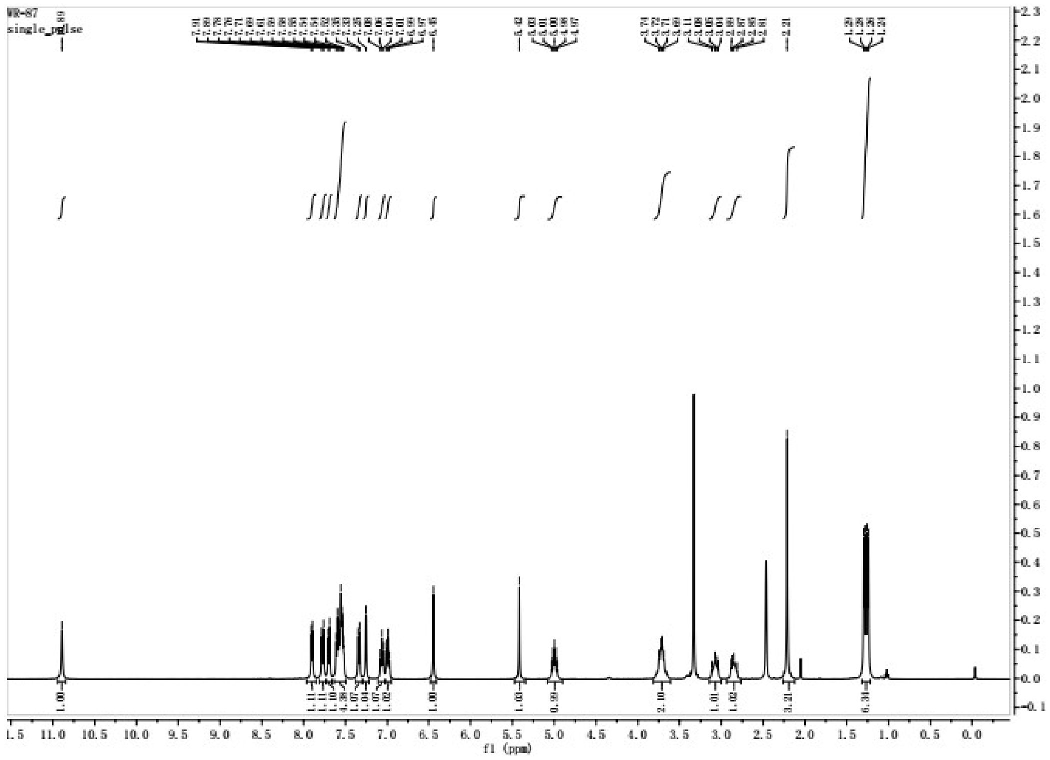

[13]C for 6c

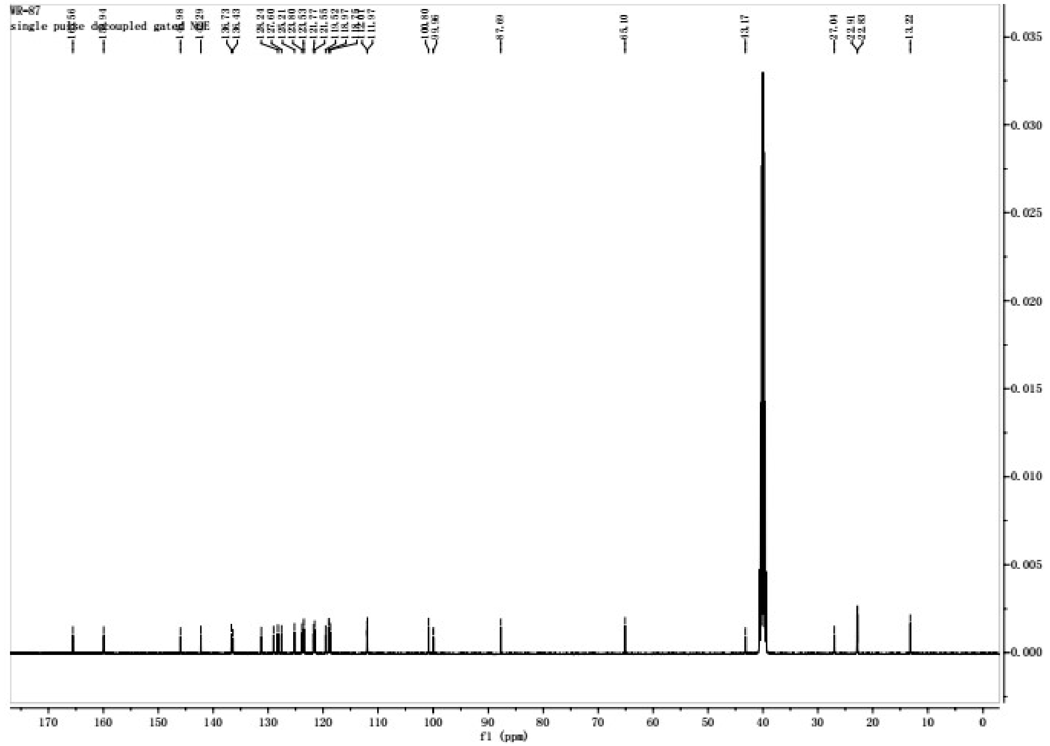

^{1}H for 6d

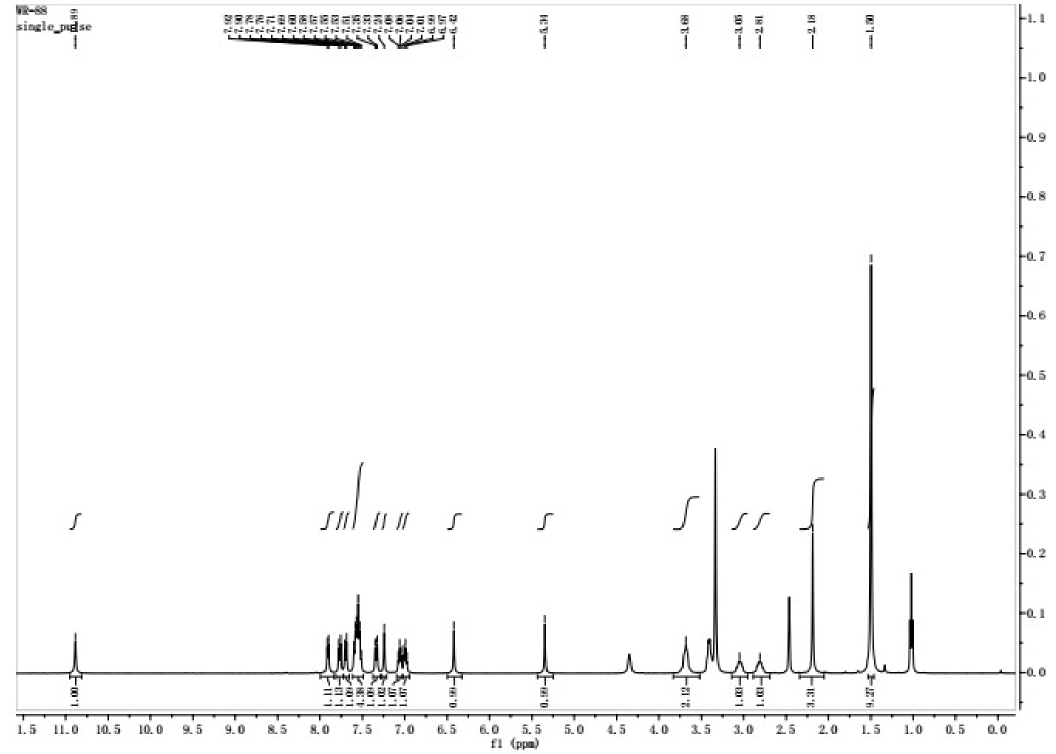

^{13}C for 6d

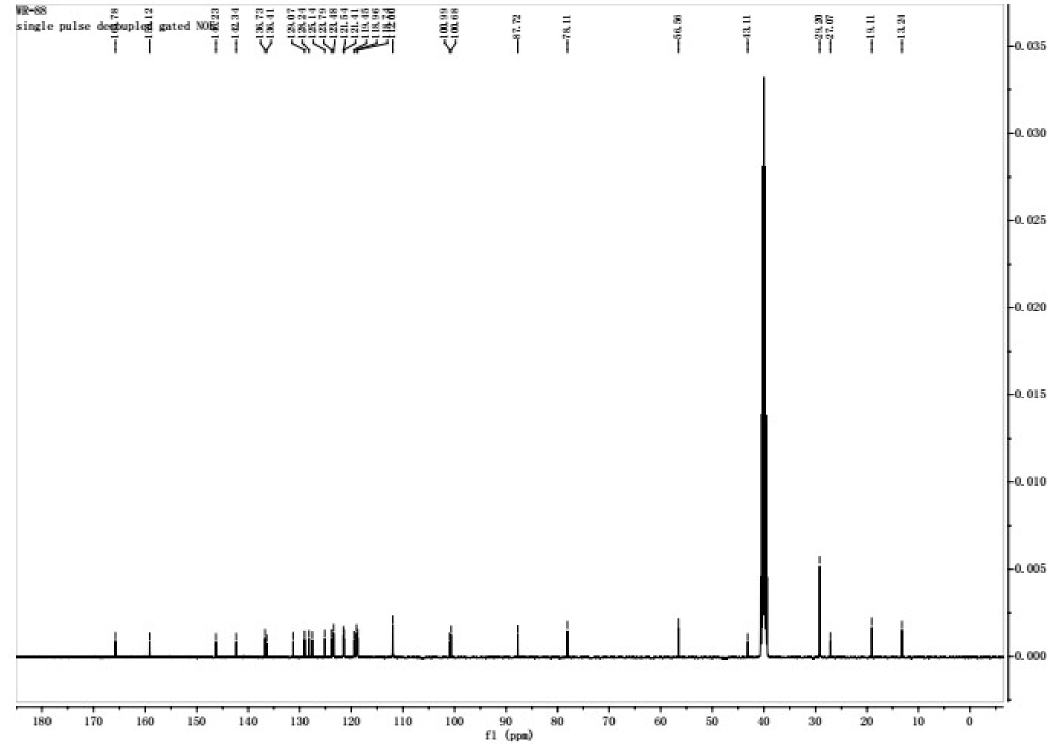

^{1}H for 6e

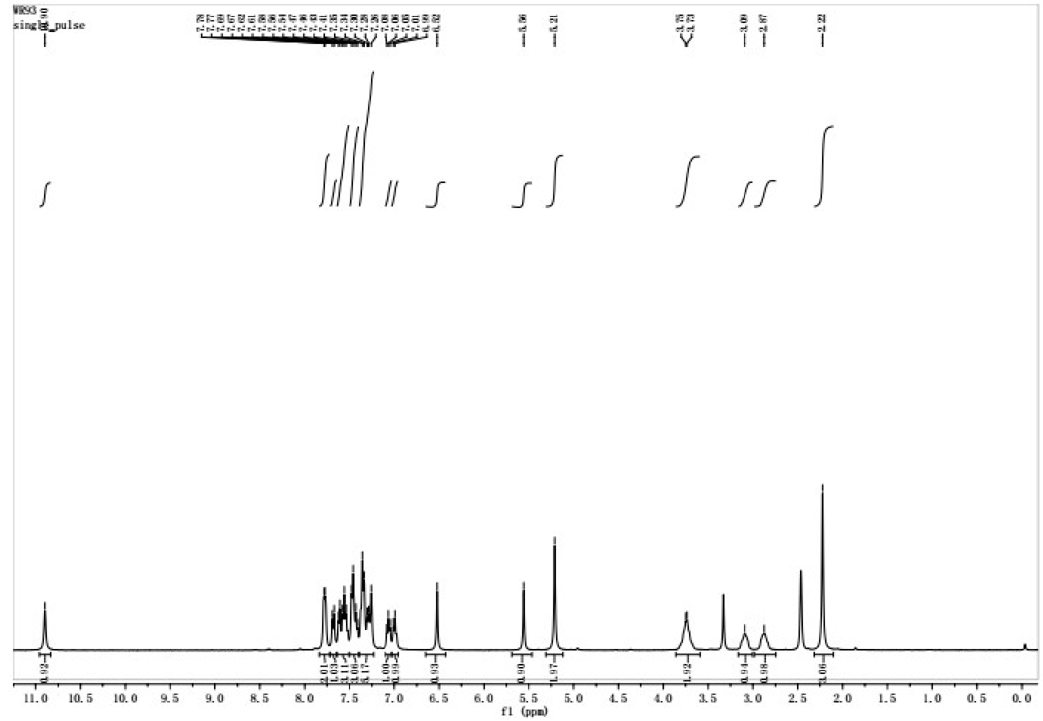

^{13}C for 6e

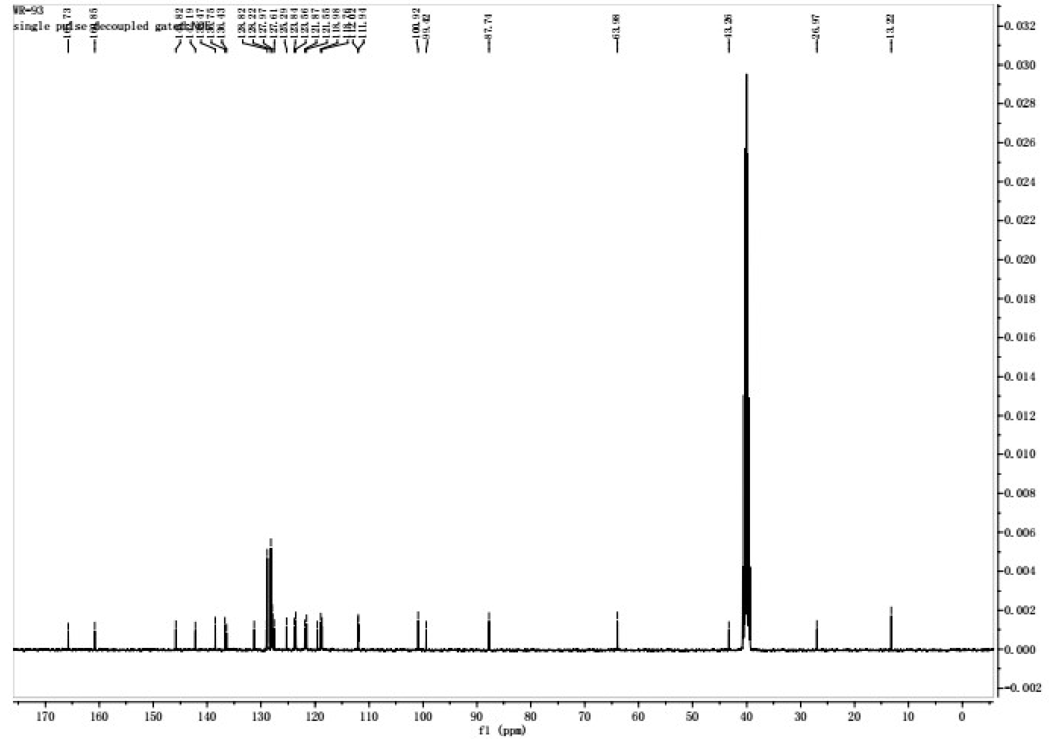

[1]H for 8a

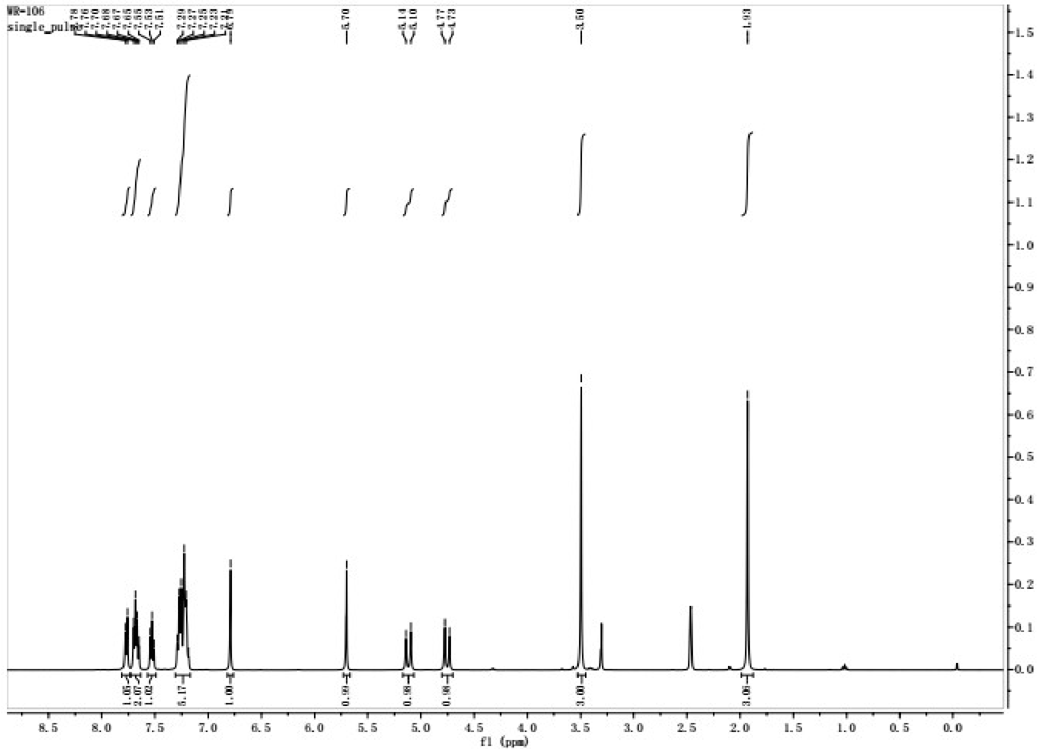

[13]C for 8a

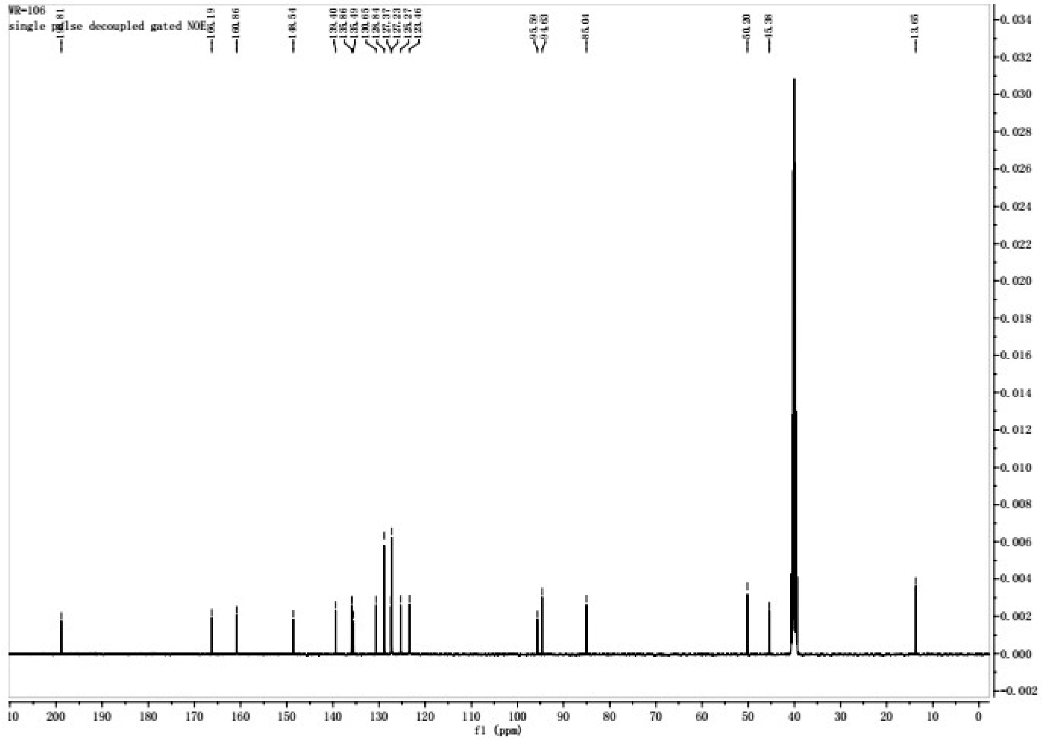

^{1}H for 8b

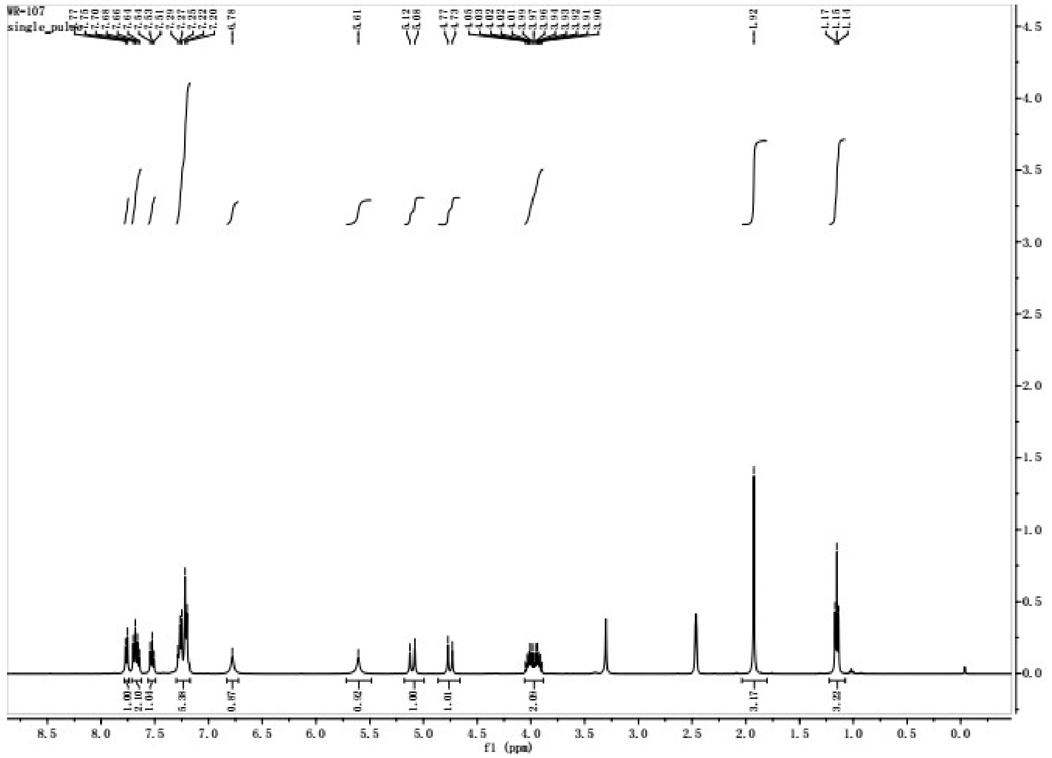

^{13}C for 8b

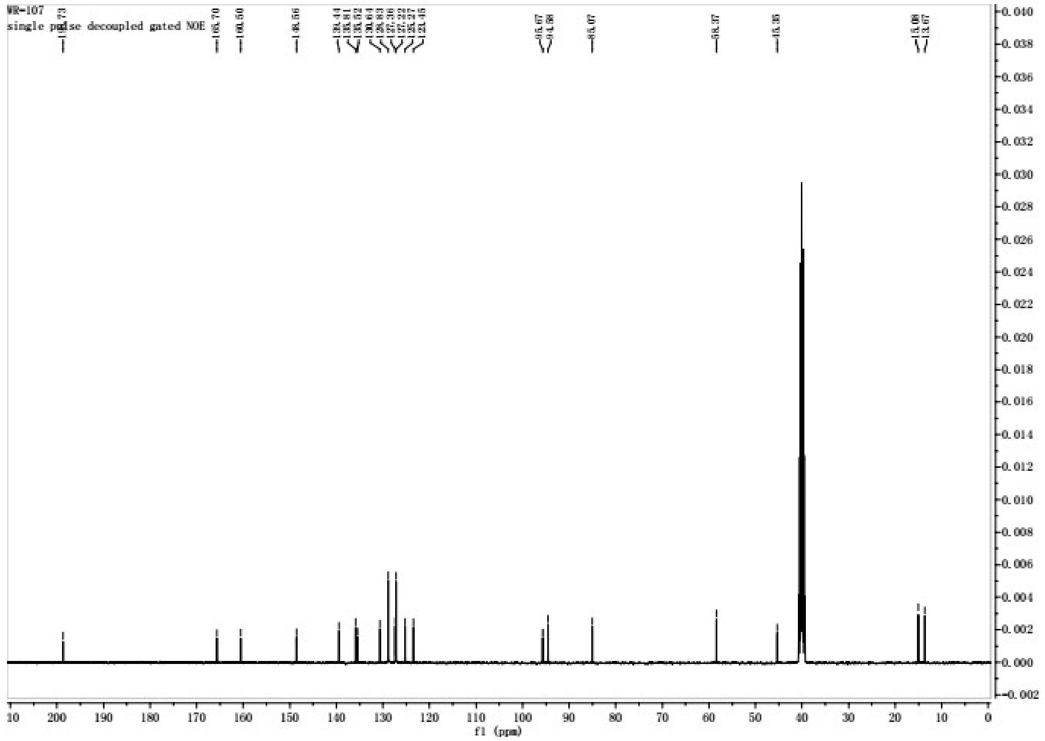

[1]H for 8c

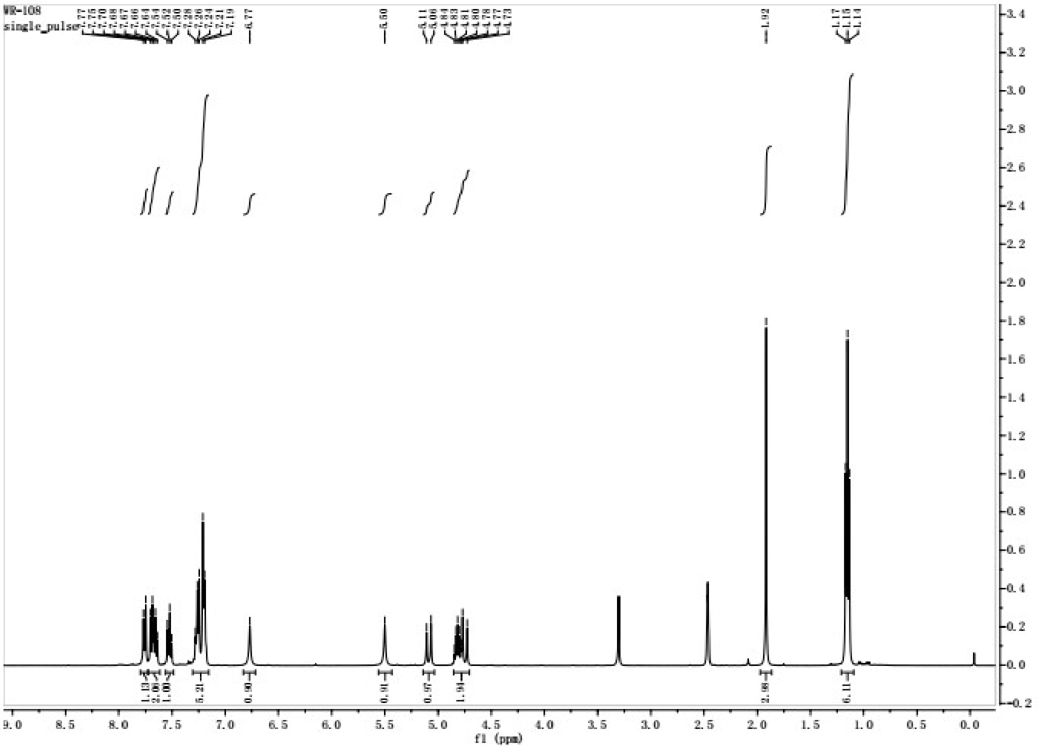

[13]C for 8c

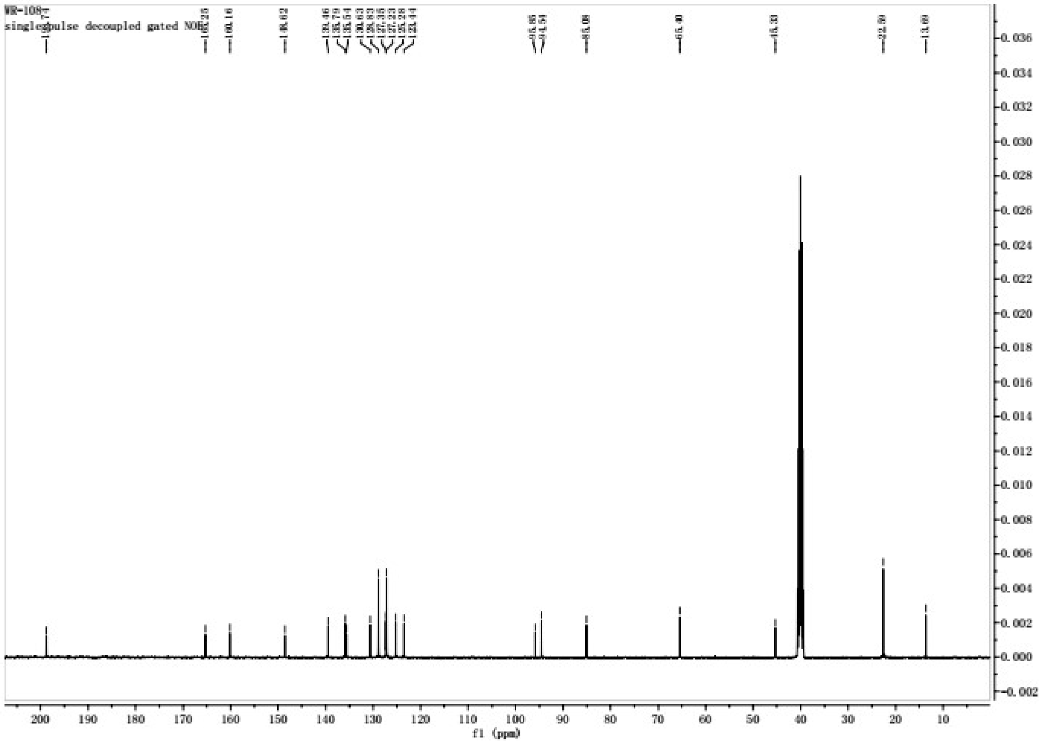

[1]H for 8d

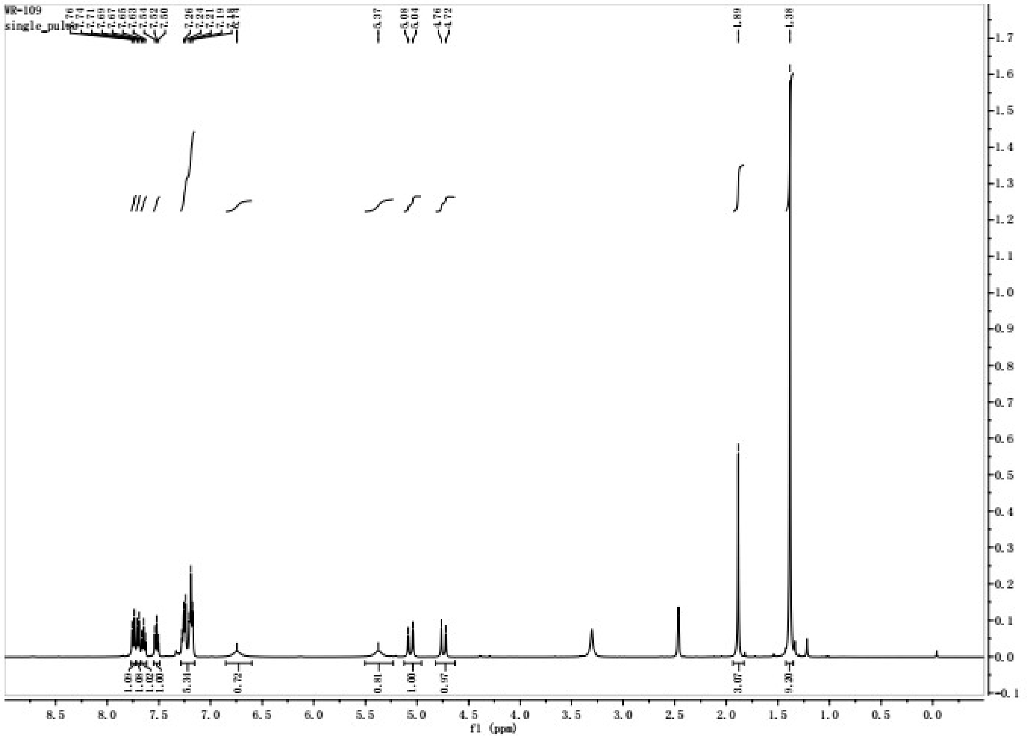

[13]C for 8d

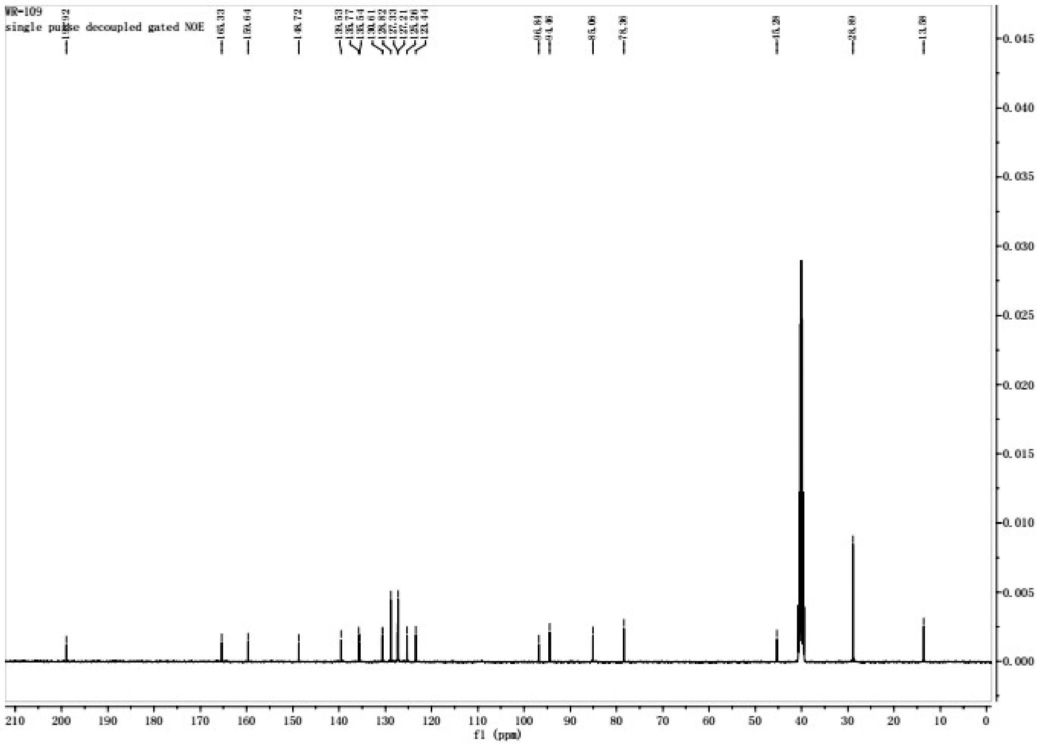

[1]H for 8e

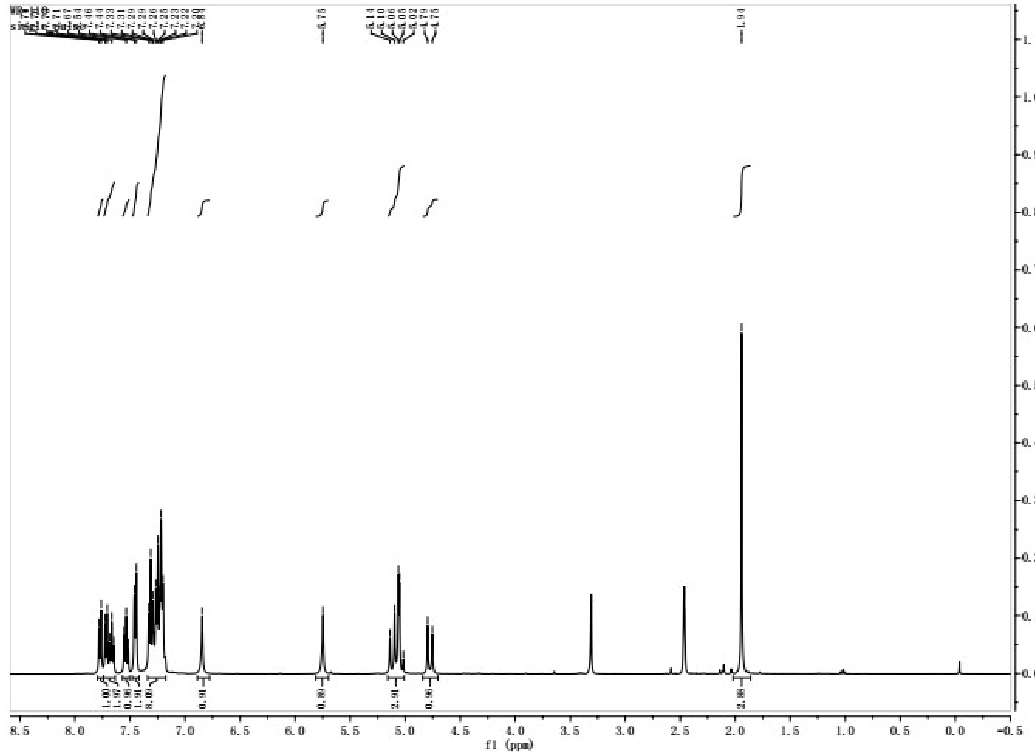

[13]C for 8e

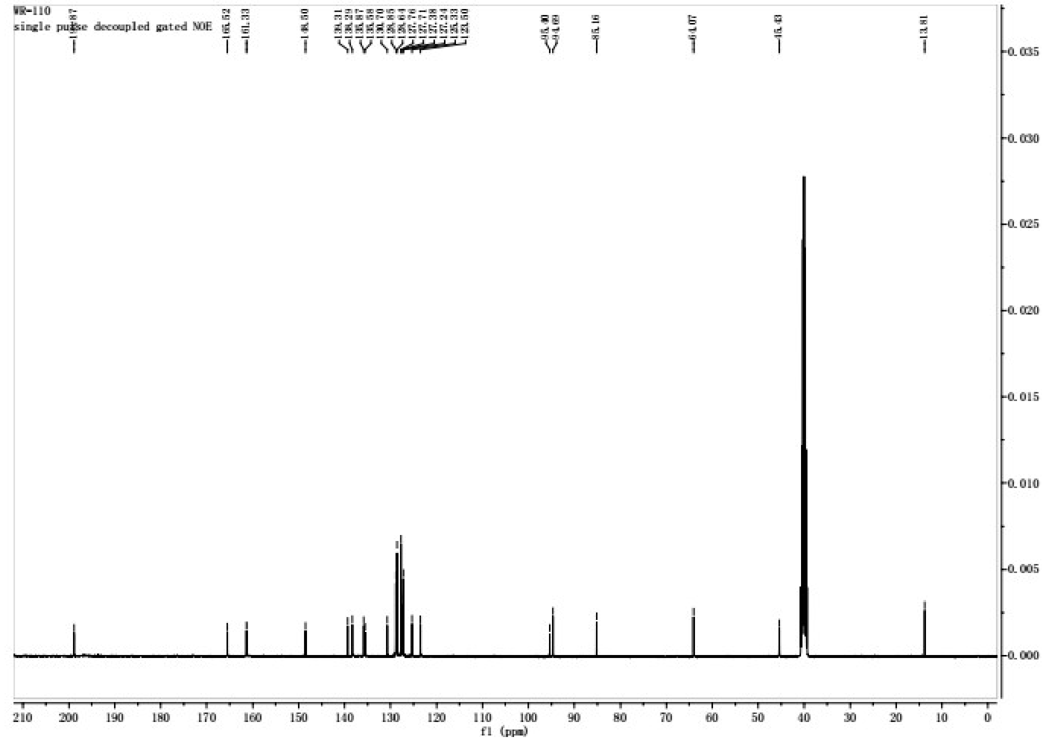

^{1}H for 8f

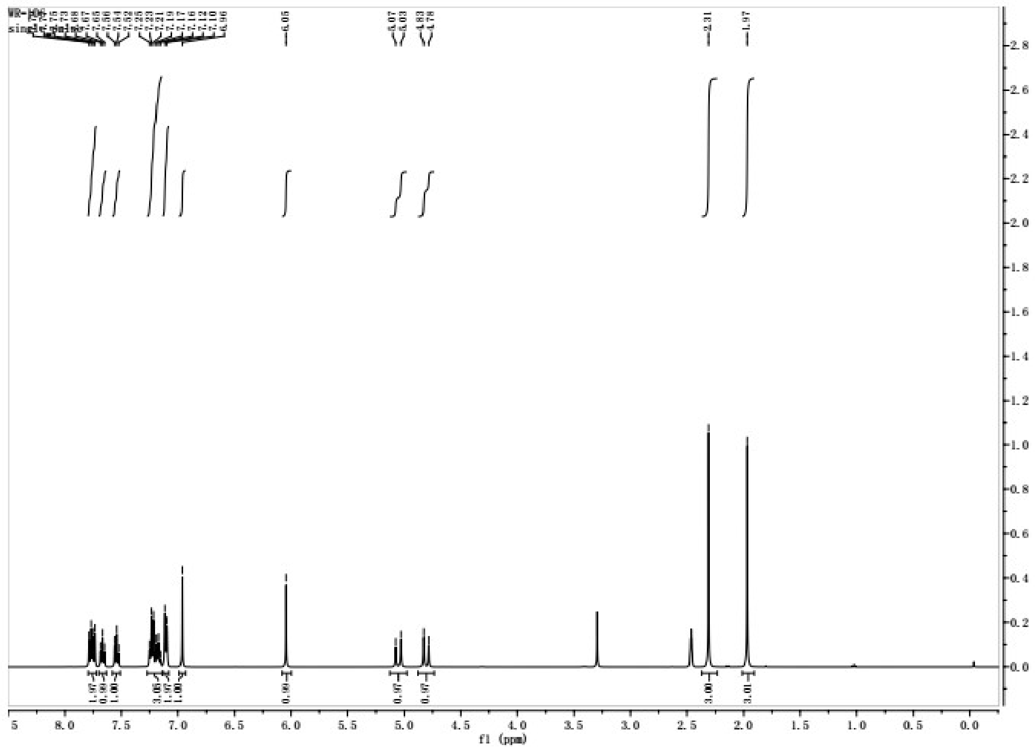

^{13}C for 8f

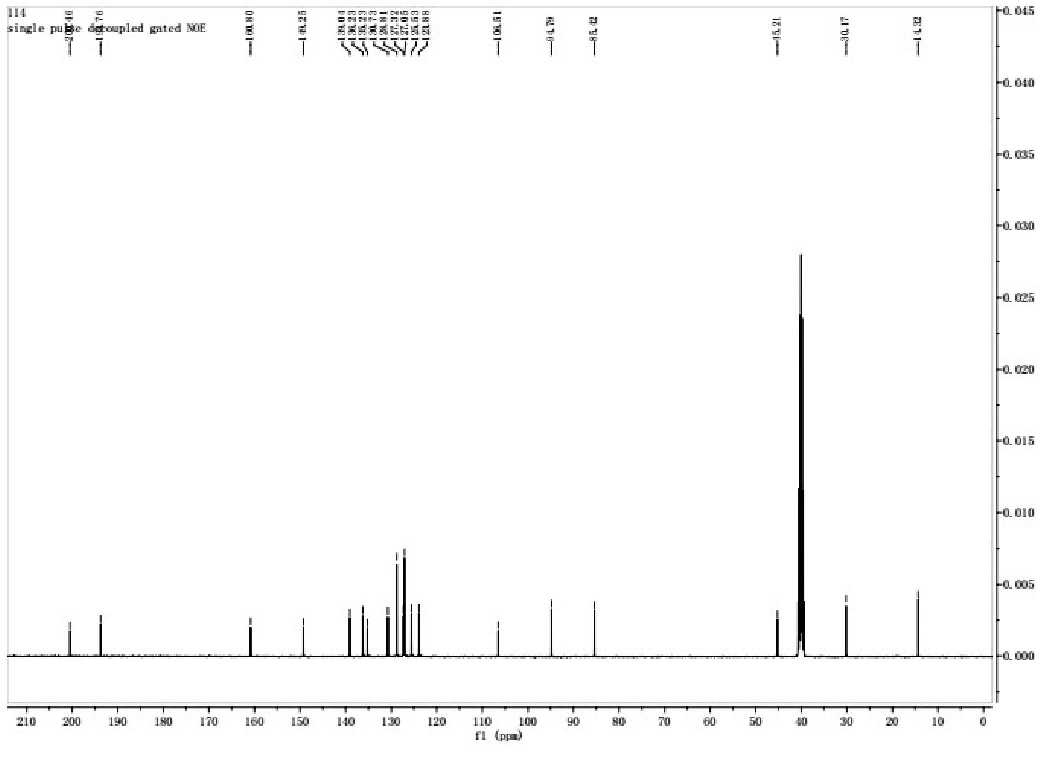

Supplement: Supplementary file 1 [file molecules-23-03031-s001.pdf]
